# A Metagenomic Meta-analysis Reveals Functional Signatures of Health and Disease in the Human Gut Microbiome

**DOI:** 10.1128/mSystems.00332-18

**Published:** 2019-05-14

**Authors:** Courtney R. Armour, Stephen Nayfach, Katherine S. Pollard, Thomas J. Sharpton

**Affiliations:** a Molecular and Cellular Biology Program, Oregon State University, Corvallis, Oregon, USA; b Department of Microbiology, Oregon State University, Corvallis, Oregon, USA; c Environmental Genomics and Systems Biology Division, Lawrence Berkeley National Laboratory, Berkeley, California, USA; d Gladstone Institutes, San Francisco, California, USA; e Department of Epidemiology & Biostatistics, Institute for Human Genetics, Quantitative Biology Institute, and Institute for Computational Health Sciences, University of California, San Francisco, California, USA; f Chan-Zuckerberg Biohub, San Francisco, California, USA; g Department of Statistics, Oregon State University, Corvallis, Oregon, USA; University of Trento

**Keywords:** arthritis, cancer, disease, humans, inflammatory bowel disease, liver cirrhosis, metagenomics, microbiome, obesity, type 2 diabetes

## Abstract

While recent research indicates that human health is affected by the gut microbiome, the functional mechanisms that underlie host-microbiome interactions remain poorly resolved. Metagenomic clinical studies can address this problem by revealing specific microbial functions that stratify healthy and diseased individuals. To improve our understanding of the relationship between the gut microbiome and health, we conducted the first integrative functional analysis of nearly 2,000 publicly available fecal metagenomic samples obtained from eight clinical studies. We identified characteristics of the gut microbiome that associate generally with disease, including functional alpha-diversity, beta-diversity, and beta-dispersion. Using regression modeling, we identified specific microbial functions that robustly stratify diseased individuals from healthy controls. Many of these functions overlapped multiple diseases, suggesting a general role in host health, while others were specific to a single disease and may indicate disease-specific etiologies. Our results clarify potential microbiome-mediated mechanisms of disease and reveal features of the microbiome that may be useful for the development of microbiome-based diagnostics.

**IMPORTANCE** The composition of the gut microbiome associates with a wide range of human diseases, but the mechanisms underpinning these associations are not well understood. To shift toward a mechanistic understanding, we integrated distinct metagenomic data sets to identify functions encoded in the gut microbiome that associate with multiple diseases, which may be important to human health. Additionally, we identified functions that associate with specific diseases, which may elucidate disease-specific etiologies. We demonstrated that the functions encoded in the microbiome can be used to classify disease status, but the inclusion of additional patient covariates may be necessary to obtain sufficient accuracy. Ultimately, this analysis advances our understanding of the gut microbiome functions that constitute a healthy microbiome and identifies potential targets for microbiome-based diagnostics and therapeutics.

## Abstract (precis)

The composition of the gut microbiome associates with a wide range of human diseases, but the mechanisms underpinning these associations are not well understood. To shift toward a mechanistic understanding, we integrated distinct metagenomic data sets to identify functions encoded in the gut microbiome that associate with multiple diseases, which may be important to human health. Additionally, we identified functions that associate with specific diseases, which may elucidate disease-specific etiologies. We demonstrated that the functions encoded in the microbiome can be used to classify disease status, but the inclusion of additional patient covariates may be necessary to obtain sufficient accuracy. Ultimately, this analysis advances our understanding of the gut microbiome functions that constitute a healthy microbiome and identifies potential targets for microbiome-based diagnostics and therapeutics.

## INTRODUCTION

Mounting evidence implicates the gut microbiome as a critical component of human health. For example, research demonstrates that gut microbiota contribute to immunity, nutrition, and behavior (1, 2). Additionally, gut microbiomes of diseased individuals tend to harbor different taxa and contain different genes than those of healthy individuals (3). These observations motivate the hypothesis that human health depends, in part, upon the taxonomic composition of and biological functions executed by gut microbiota. Accordingly, researchers have sought to identify the properties of the human gut microbiome that signify health and disease. Such signatures are valuable to resolve because they provide important context for the development of disease diagnostics, clarify disease etiology, and generate insight into how microbiomes could be amended to restore health.

Prior investigations focused on defining how the gut microbiome signifies health or disease. For example, the Human Microbiome Project defined the structure and function of the gut microbiome in clinically healthy, urban North Americans (4). Other investigations used clinical 16S rRNA gene sequence data to determine how the structure of the gut microbiome of diseased individuals differs from that of healthy individuals (3, 5). More recently, a smaller set of investigations used shotgun metagenomes to resolve how both the structure and functional diversity of the gut microbiome associate with disease (6 – 13). However, almost all prior investigations focused on a single disease population and a matching control. Very few studies integrate data across multiple populations, incorporate data from other studies, or compare patterns across various disease types. Consequently, it is unclear which associations are robust to population or study effects. Moreover, we possess limited insight into which associations are specific to a disease type versus those that are common to myriad diseases. These limitations hinder our ability to develop robust clinical diagnostics from microbiome data and obscure our understanding of the potential mechanisms through which the microbiome contributes to a specific disease or health in general.

Integrating data across investigations through a meta-analysis overcomes these limitations (14 – 16). Though their application in microbiome science remains limited, meta-analyses provide important clarity in microbiome research. For example, meta-analysis of 16S rRNA gene sequence-based investigations surrounding human obesity revealed that originally reported associations between the taxonomic composition of the gut microbiome and obesity were inconsistent across studies (17) and appear to manifest only weak statistical effects (18). Additionally, a meta-analysis of 16S rRNA gene sequence data quantified the microbiome’s taxonomic association with disease across several populations that span a variety of diseases to reveal that some microbiome characteristics are disease specific while others are common to multiple diseases (15). The application of meta-analyses to shotgun metagenomic data is even more restricted, in part due to the limited amount of clinical metagenomic data currently available. One study integrated metagenomes to assess the predictive capacity of the taxonomic profile of the microbiome for several diseases, finding that integrating multiple data sets improved prediction capabilities (16). These studies highlight the importance of data integration in contributing to our understanding of the role of the microbiome in health and disease.

While these studies have proven insightful, their focus on taxonomy may limit our understanding of how the microbiome relates to health. Metagenomes afford insight into the types of genes contained, and consequent biological pathways encoded, by the microbiome. Resolving the association between microbiome functions and health may prove critical to determining the mechanisms through which the microbiome promotes health or contributes to diseases. Moreover, such analyses may reveal robust indicators of disease given observations that different microbes can elicit analogous functional effects on the host (19, 20). For example, the application of meta-analysis to the functional diversity of the gut microbiome in a study of type 2 diabetes revealed gene families contained in the microbiome that consistently associate with disease across two continents (21). The integration of metagenomic data sets in this study revealed the confounding contribution of antidiabetic medicine to the results, emphasizing the need to consider additional factors, such as medication, in assessments of the gut microbiome’s relationship to health and disease.

Here, we describe the first meta-analysis of microbiome gene functions that spans multiple disease types and populations. For this meta-analysis, we identified all publicly available human shotgun metagenomic microbiome data with diseased and nondiseased subjects, which consist of ∼2,000 metagenomes that span 8 studies and 7 diseases. We selected a case and control population for each disease from the available samples and applied a regression-based statistical framework to assess how the functional capacity of the microbiome varies in association with each disease and across diseases in general. Where possible, we modeled data spanning multiple studies with a study variable to control for potential study effects. Our study (i) reveals that functional diversity indicates disease, but usually with weak effect; (ii) resolves microbiome functions that associate with multiple diseases as well as functions that indicate specific diseases; (iii) documents the importance of considering study-specific parameters when deriving diagnostics based on the functional diversity of the gut microbiome; and (iv) explores the ability of the functional composition to predict disease status.

## RESULTS

### Gut metagenome functional diversity associates with disease.

**FIG 1:**
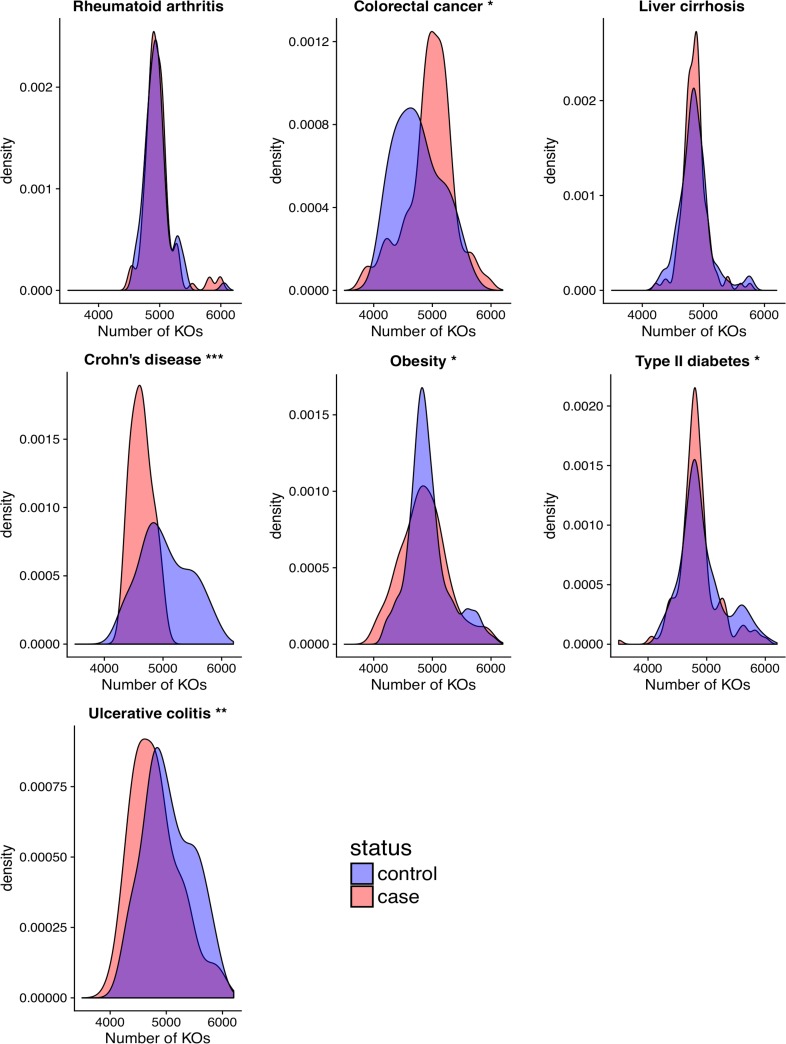
Protein family richness associates with disease. Density plots of the distribution of protein family richness across case and control populations for the seven diseases. Asterisks beside plot titles indicate significance from Student’s *t* test (*, *P* < 0.05; **, *P* < 0.01; ***, *P* < 0.001). Similar results were observed with Kolmogorov-Smirnov and Kruskal-Wallis tests.

After preprocessing the data (Materials and Methods), we statistically integrated publicly available gut metagenomic data from 1,473 patients spanning seven diseases and eight studies to discern how the functional diversity of the gut microbiome associates with disease. In particular, we investigated how gut microbiome protein family richness, composition, and dispersion relate to disease. Our analysis of gut metagenome protein family richness revealed that patients diagnosed with Crohn’s disease (*P* < 0.001), obesity (*P* < 0.05), type 2 diabetes (*P* < 0.05), or ulcerative colitis (*P* < 0.01) manifest a reduced number of KEGG Orthology Groups (KOs) compared to their respective control populations (Fig. 1). Conversely, subjects with colorectal cancer harbored a larger number of microbiome protein families than their controls (*P* < 0.01). The protein family richness in the microbiome of subjects with liver cirrhosis or rheumatoid arthritis was similar to their respective controls. With the exception of type 2 diabetes, these results were robust to rarefaction.

**FIG 2:**
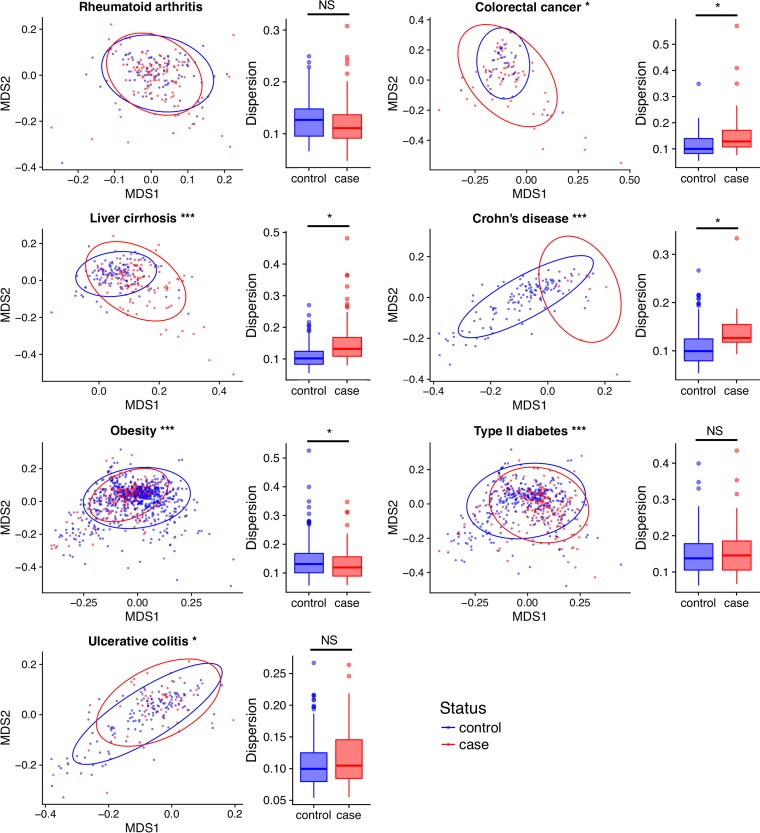
Changes in functional composition associate with disease. NMDS plots of Bray-Curtis dissimilarity between cases and controls across diseases; ellipses represent 95% confidence level. Asterisks in NMDS plot titles indicate significance from PERMANOVA (***, *P* < 0.001; Table S6). Box plots represent dispersion in beta-diversity within groups. Asterisks in box plots denote significance from *P* test and ANOVA (*, *P* < 0.05).

To determine how the functional composition of the gut metagenome relates to disease, we quantified the Bray-Curtis dissimilarity between all samples based on their KO abundances. Our analysis found that the functional composition of the gut microbiome differs between case and control populations for the following six diseases: colorectal cancer, liver cirrhosis, Crohn’s disease, ulcerative colitis, obesity, and type 2 diabetes (Adonis *P* < 0.05; Fig. 2; see also Table S2 in the supplemental material). However, the magnitude of these differences varied across diseases (Table S2), ranging from relatively strong effects in Crohn’s disease (partial *R*^2^ = 10.3%) to weak effects in obesity (partial *R*^2^ = 1.2%). Meanwhile, rheumatoid arthritis exhibited no detectable differences in functional composition between cases and controls.

Since disease status tends to explain a small proportion of the variation in microbiome functional composition, we modeled available covariates to identify additional factors that contribute to the variation in functional composition. The additional metadata common to most subjects in these studies were limited to age, BMI, sex, country, and study. Each of these variables associated with the composition of the gut microbiome in previous research (22 – 25). We found that age comprised between 0.4% and 9.3% variation (*P* < 0.05; Table S2) across all diseases except arthritis and liver cirrhosis, while sex significantly contributed variance only to liver cirrhosis (1.64%, *P* < 0.001) and obesity (0.63%, *P* < 0.01). After accounting for disease status, BMI significantly associated only with colorectal cancer (2.57%, *P* < 0.0.5).

The obesity and type 2 diabetes populations that we analyzed comprise individuals from different studies, which affords an opportunity to measure how variation across studies (e.g., technical variation) affects measures of the microbiome’s functional association with disease. We found that, at least for obesity and type 2 diabetes, study accounts for 18.1% and 14.9%, respectively, of the variation in beta-diversity (Table S2). However, study and country confound one another since most studies included in our analysis sampled patients from distinct countries. While technical variation is thought to impact gut microbiome composition (26), a previous study found that the variation introduced by these factors (e.g., library size, read length, and quality control parameters) is relatively small in contrast to biological variation between samples, indicating that these technical factors are unlikely to influence our results (27). Additionally, we found that DNA extraction protocol explains relatively little variance in the functional composition of the gut microbiome relative to study or geographic region (Text S1). Consequently, we concluded that the observed variation between studies likely reflects geographic structure in how the microbiome relates to disease.

10.1128/mSystems.00332-18.1 TEXT S1 Text file describing methodology and results of analyses on the contribution of DNA sequencing method to variance in gut microbiome module abundance and comparison of regression results of the merged data sets to individual data sets for obesity and type 2 diabetes. Download Text S1, PDF file, 0.5 MB. Copyright © 2019 Armour et al. 2019 Armour et al. This content is distributed under the terms of the Creative Commons Attribution 4.0 International license.

Beta-dispersion measures the compositional variation of the microbiome among a group of individuals, and prior work linked disease to an increase in taxonomic beta-dispersion (28). We similarly measured whether gut microbiome functional beta-dispersion varies between healthy and diseased populations. We observed an increase in functional beta-dispersion among individuals diagnosed with colorectal cancer, Crohn’s disease, and liver cirrhosis (*P* < 0.05; Fig. 2). Individuals afflicted with obesity displayed reduced beta-dispersion relative to their controls. The remaining diseases presented no detectable difference in functional beta-dispersion. As observed with functional richness and beta-diversity, the effect size of beta-dispersion varied across diseases but for some diseases appeared to be relatively substantial.

### Metagenome modules indicate disease and clarify mechanisms of health.

We next examined whether specific microbiome functions associate with disease. To reduce data dimensionality, we collapsed KOs into modules. We then used compound Poisson linear regression to model the relationship between health state and the average genomic copy number of KEGG modules. These methods were applied in prior work (29) and allow for robust modeling of sparse but otherwise continuous data. Moreover, they afford the ability to account for potential study effects through the inclusion of additional covariates. Where possible, we incorporated study effects into our models and identified indicators of disease that were robust to study effects. Using these models, we defined indicators of a disease to be those modules whose average genomic copy number in the metagenome significantly associated with the health status of the host. While we were able to model study effect in our discovery of indicators, it is unclear how robust study indicators are across distinct studies in part due to substantial variation in study size (Text S1). Most likely, not all indicators will be robust across populations; to identify robust disease indicators across populations, data from additional clinical studies on the same diseases are needed.

We found that of the 521 modules defined across our data set, 484 indicated disease in one or more of the disease populations (FDR < 0.2). The number of modules that indicated disease varied considerably across diseases (Tables S3 and S4). For example, 333 and 349 modules indicated liver cirrhosis and Crohn’s disease, respectively, while only 13 modules indicated ulcerative colitis. These results were qualitatively consistent at lower FDR thresholds as well as at the KO level (Table S4).

**FIG 3:**
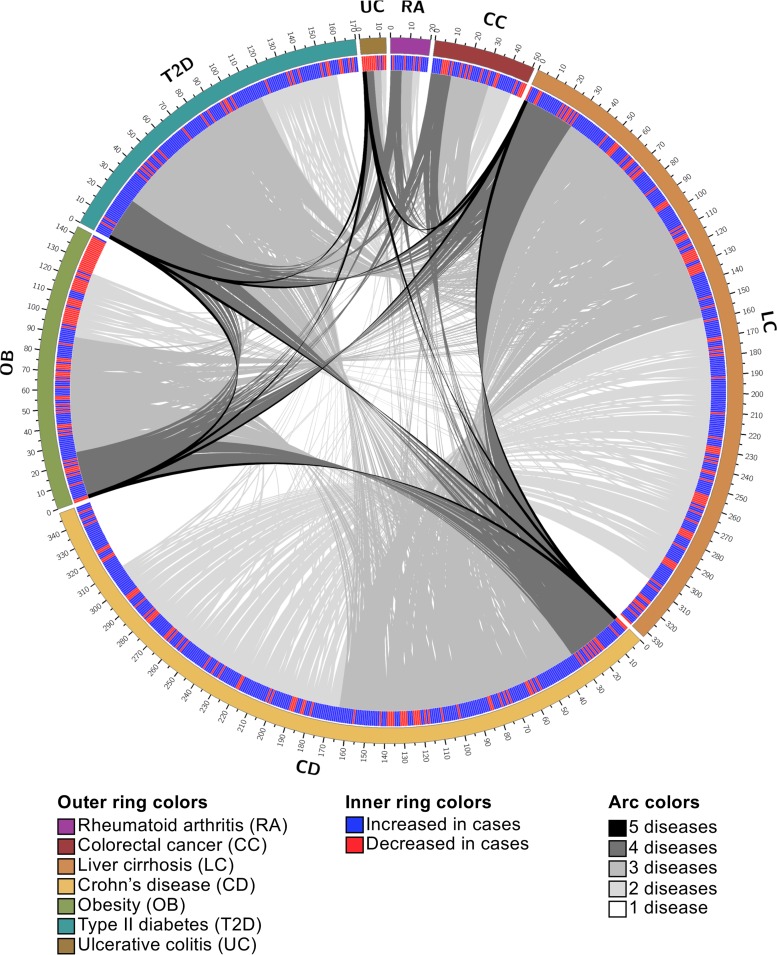
Most indicators of disease are shared. Circos plot depicting overlap of indicator modules between diseases. The outer track represents the total number of module markers for each disease. The second track is a heat map with blue representing an increase in cases and red representing a decrease in cases. Modules in each disease are ordered and links are colored by the number of diseases for which they are indicators (black, 5 diseases; dark gray, 4 diseases; medium gray, 3 diseases; light gray, 2 diseases; white, 1 disease). Modules without links are unique to the given disease.

The vast majority of the disease-indicating modules acted as indicators for multiple diseases. Specifically, 78% of the indicator modules associated with two or more diseases, which is higher than expected if indicator modules were randomly distributed among diseases (permutation test, 1,000 permutations, *P* < 0.05). There were relatively few unique indicator modules for each disease; for example, only 7.5% and 11.2% of indicator modules were unique to liver cirrhosis and Crohn’s disease, respectively (Fig. 3 and Table S5). These results suggest that different diseases may manifest similar mechanisms of association with the gut microbiome (e.g., inflammation), that microbiome modules may play various roles in determining how the microbiome associates with different diseases, or that there is a factor common to both disease and changes in the gut microbiome (e.g., lifestyle).

We reasoned that the high frequency of modules that indicate multiple diseases may reflect the existence of modules that indicate any disease. While no modules stratified cases and controls across all seven diseases in our analysis, 33 modules indicated at least four but no more than five distinct diseases, and each disease is indicated by at least six of these 33 common disease indicators (Table S5). Some diseases are indicated by a large proportion of these modules, including Crohn’s disease (97%), liver cirrhosis (88%), type 2 diabetes (73%), and obesity (79%). Conversely, rheumatoid arthritis (18%), colorectal cancer (33%), and ulcerative colitis (18%) are indicated by relatively few modules. That said, these modules can constitute a substantial fraction of the total indicators discovered for the latter set of diseases, as evidenced by the fact that 46% of the ulcerative colitis modules are among this common set. Despite the frequent number of diseases indicated by these modules, they do not always indicate diseases through consistent signatures. For example, N-glycosylation by oligosaccharyltransferase (M00072), which happens to indicate the largest number of diseases, is consistently depleted in individuals affected by liver cirrhosis, Crohn’s disease, obesity, type 2 diabetes, and ulcerative colitis relative to controls. Conversely, modules for cobalt/nickel transport systems (M00245 and M00246) are depleted in subjects with colorectal cancer and Crohn’s disease but elevated in subjects with liver cirrhosis and type 2 diabetes. Additional common indicators include modules associated with lipopolysaccharide biosynthesis and export (M00060, M00320, and M00080), iron/zinc/manganese/copper transport system (M00318), and acetate production (M00422, M00377, M00618, and M00579). These results demonstrate that while no microbiome modules universally signify health, there exist modules that are commonly perturbed during disease.

The relatively small number of modules that uniquely indicate disease provide insight into disease etiology and advance the development of disease-specific diagnostics. Diseases varied in the proportion of their indicators that uniquely define the disease. For example, 20% of the rheumatoid arthritis indicators are unique while only 7% of type 2 diabetes indicators are unique. This observation highlights the fact that some diseases may offer greater potential for the discovery of microbiome-based clinical diagnostics. In rheumatoid arthritis subjects, the unique indicators include elevated levels of modules associated with methane production (M00617) and a DevS-DevR two-component regulatory system (M00482) that associates with Mycobacterium tuberculosis virulence (30). The microbiomes of colorectal cancer subjects have increased abundance of a module for naphthalene degradation (M00534). The modules increased in the microbiome of liver cirrhosis subjects include nitrification (M00528) and staphylococcal virulence regulation (M00468). In contrast, there is a decrease in a module for toluene degradation (M00418). In Crohn’s disease, the modules increased in the microbiome of cases include degradation of glycosaminoglycans (M00076, M00077, M00078, and M00079) and B-vitamin biosynthesis (M00122, M00123, and M00573). There is a decrease in abundance of modules associated with methanogenesis (M00576), antimicrobial peptide response (M00470), and phosphatidylethanolamine biosynthesis (M00092). The unique modules in the microbiome of obesity subjects include enterohemorrhagic Escherichia coli (EHEC) pathogenicity signature (M00363). Type 2 diabetes cases have an increase in nitrogen fixation (M00175), glutamate transport (M00233), and capsaicin biosynthesis (M00350) and a decrease in O-glycan biosynthesis (M00056) in their gut microbiomes. There are not any unique indicators for subjects with ulcerative colitis.

It is possible that some functional indicators identified in this analysis are not directly connected to disease but are genomically linked to functions that are themselves connected to disease. Such hitchhiking indicators are, in effect, the result of a taxon’s association with disease. To identify which taxa may drive the differential abundance of functional indicators observed in our analyses, we taxonomically annotated each metagenome using MetaPhlAn2 (31) and correlated the relative abundance of each functional indicator (*n* = 484 indicator modules) with each observed taxon. This analysis revealed significant genus-module correlations for 422 indicator modules of at least one disease (FDR < 0.05, Spearman’s |ρ| > 0.4; Fig. S2A to G and Table S6), while 62 of the 484 indicator modules elicited no significant genus associations. The observed associations were distributed across 93 genera, with *Subdoligranulum, Bacteroides, Prevotella, Escherichia, Methanobrevibacter, Blautia,* and an unknown genus within the *Clostridiales* manifesting a disproportionately large number of associations relative to the interquartile range of taxon-module association frequencies. These taxa may carry a large number of indicators in their genomes or alternatively are themselves ecologically linked to the presence of taxa that encode indicator modules. Only 37 modules correlate with a single genus, indicating that multiple taxa may contribute to the signal of differential abundance of functional indicators in the metagenome or that functional indicators ecologically link to an array of microbial taxa. The potential for closely related taxa to contain different functional repertoires reinforces the importance of considering the microbiome not only through a taxonomic perspective but also in terms of the functions present.

### The functional composition of the microbiome can classify disease status.

**FIG 4:**
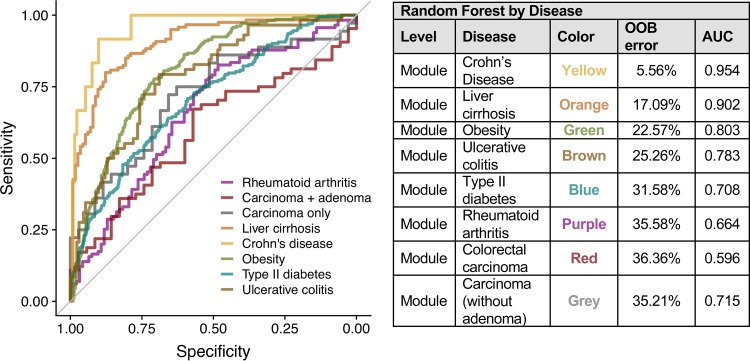
Classifying disease status based on the functional composition of the microbiome. ROC curves from random forest classifiers for cases and controls in each disease. The table shows OOB error and AUC values.

Each disease examined in this analysis has a set of indicator modules that stratify cases and controls, prompting the question of whether the functional composition of the gut microbiome can be used to classify disease status. To answer this question, we implemented a random forest machine learning approach to diagnose an individual’s disease status for each disease based on microbiome module abundance. We found that the classification sensitivity and specificity vary as a function of disease (Fig. 4), where cases and controls were accurately resolved for some diseases (e.g., Crohn’s disease, area under the curve [AUC] = 0.9539, and liver cirrhosis, AUC = 0.9023) but not others (e.g., rheumatoid arthritis, AUC = 0.6641, and colorectal cancer, AUC = 0.5955). The low AUC for colorectal cancer is inconsistent with prior results classifying colorectal cancer subjects based on microbiome module abundance (32 – 34). This discrepancy in classifier performance is due to differences in the selection of cases and controls: the prior studies excluded subjects with precancerous adenomas while our classifier included them as cases. Exclusion of the advanced adenoma subjects results in improved classifier performance similar to the prior study (AUC, 0.72; Fig. 4). These results suggest that the potential for use of the functional composition of the gut microbiome in disease diagnosis varies by the type and severity of disease.

As noted above, a majority of modules defined in this data set stratify cases and controls in at least one disease (484 out of 521). Additionally, most of these differentially abundant modules are indicators for more than one disease. Due to this overlap in indicator modules, we postulated that the module abundance could classify subjects into diseased versus nondiseased groups, regardless of the disease. We found that module abundance has moderate ability to predict diseased or nondiseased status (AUC = 0.6738, out of bag [OOB] = 39.04%). This suggests that there may not be enough similarities across these diseases to classify diseased individuals from controls.

## DISCUSSION

Our integrative analysis reveals the functional attributes of the gut metagenome that relate to human health and disease. We show that healthy microbiomes tend to encode higher protein family richness, significantly different functional compositions, and increased constraint on the variation in that composition compared to disease-associated microbiomes. However, effect sizes are frequently weak and not all diseases manifest these trends. Moreover, we identify specific functional modules that associate broadly with disease and, therefore, may be important to maintaining host health. Additionally, we resolve disease-specific markers that help clarify disease etiology and assess the ability of potential biomarkers to classify health status. Ultimately, the microbiome functions that we identify as being enriched in healthy individuals and disrupted in diseased individuals may illuminate how the microbiome contributes to host health.

Disease tends to associate with a reduction in the number of distinct protein families encoded in the microbiome. However, this trend is not universal, where some diseases (i.e., liver cirrhosis and rheumatoid arthritis) have no significant difference in richness and others (i.e., colorectal cancer) exhibit an increase in richness in diseased subjects. Decreased taxonomic richness commonly associates with disease, and some studies have associated decreased functional richness with disease (35). While this holds true for several diseases (i.e., Crohn’s disease, obesity, type 2 diabetes, and ulcerative colitis), it is not a ubiquitous characteristic of the microbiome in a diseased subject.

The integration of metagenomic data enabled comparison of the differences in the gut microbiome’s functional composition across a variety of diseases. We find that while the microbiome’s functional composition associates with host health, the strength of the association substantially varies by disease and is generally relatively small. This suggests that these diseases are not defined by a substantial restructuring of the functional composition of the gut microbiome. Rather, if the microbiome contributes to diseases, it tends to do so through changes in the abundance of specific protein families, which may be different in each diseased subject. Consequently, health is not necessarily defined by the sum total of the functional capacity of the microbiome.

Among the many complexities of the gut microbiome is the variation in functional composition observed even in healthy populations that can be attributed to factors unique to a population (e.g., their geographic location) or investigation (e.g., how samples were processed). These factors may impact the apparent relationship between the microbiome and health state. These so-called study effects may thus potentially confound the discovery of microbiome signatures that robustly indicate disease, especially when data that are collected from only a single population or investigation are used to uncover these indicators. However, no investigation has yet measured how study effects impact discoveries that result from associating the microbiome’s functional diversity with health state. To date, only colorectal cancer, obesity, and type 2 diabetes have been investigated using clinical shotgun metagenomic data that were generated from multiple, distinct populations and research studies. Integrating data across these studies, we find that study accounts for approximately 18.14% and 14.92% of the variation in functional composition between cases and controls for obesity and type 2 diabetes, respectively, while disease status accounts for only 1.2% and 1.7%, respectively. This finding aligns with prior observations of study effects in analyses of the taxonomic composition of the gut microbiome (15, 26, 36).

The phrase “study effects” is an umbrella term often used to describe any unknown source of variance. Comparison of the technical and biological replicates in this data set reveals that the variation between these replicates is less than the variance between unrelated samples, indicating that certain study effects (i.e., batch effects) are unlikely to be the source of variance between samples. The variance in functional composition is more reasonably due to factors associated with geographical location such as diet and cultural practices. Unfortunately, we do not currently possess the appropriate data set to address this question. Future studies should seek to generate metagenomic data from more diverse populations that span distinct countries. Despite the large contribution of study effects, disease status remains an important factor in explaining the variance between samples.

Analysis of the microbiome’s functional beta-dispersion reveals that most diseases have increased intersample variation in the microbiomes of the case populations relative to the microbiomes of the control populations. This pattern of increased dispersion in disease-associated microbiomes was previously observed in studies of taxonomic diversity and dubbed the Anna Karenina principle (AKP) (28). AKP hypothesizes that certain stressors elicit stochastic effects on the taxonomic composition of the microbiome to yield increased variation in the stressed group relative to the control group. Our beta-dispersion analysis shows that the AKP also applies to the functional profiles of the gut microbiome in diseased hosts. This observation indicates that the increased dispersion observed in the taxonomic analysis of diseased microbiomes is unlikely to be the result of redundant functional compositions across communities, since if that were the case we would expect to find little to no increase in dispersion in the functional profiles. That said, our observation does not preclude the possibility that different taxa encode a small set of redundant proteins that associate with the disease state. For example, several genera within the phylum *Proteobacteria* (e.g., *Escherichia*, *Pantoea*, and *Sutterellaceae*) appear to contribute to the abundance of lipopolysaccharide (LPS) biosynthesis and transport modules. Additionally, our finding that there tends to be lower functional dispersion among healthy individuals indicates that there may exist greater constraints on how the microbiome operates among healthy individuals.

Our robust and integrative modeling approach reveals specific associations between microbiome function and health by identifying commonly perturbed functions that impact host health. Interestingly, most of the common indicators (i.e., indicators of four or more diseases) are increased in abundance in the microbiomes of diseased subjects relative to the microbiomes of control subjects, suggesting that these shared disease associations may be due to the elevated presence of some microbiome functions rather than their loss in the microbiome. For example, subjects with colorectal cancer, liver cirrhosis, Crohn’s disease, and obesity have increased abundance of a module for lipopolysaccharide (LPS) biosynthesis (M00060). LPS is a well-known proinflammatory molecule; increased LPS biosynthesis by gut microbiota could contribute to intestinal inflammation observed in subjects with these diseases. Additionally, some common indicators may clarify collective features of the intestinal environment across disease. For example, several modules for iron transport (M00318, M00190, M00240, M00243, M00317, and M00319) are increased in the microbiomes of subjects with Crohn’s disease, liver cirrhosis, obesity, and type 2 diabetes. Iron is an important cofactor for both humans and microbes and is often the subject of conflict between host and pathogen (37). Another common indicator is acetate production (M00377 and M00618), which is increased in the gut microbiome of subjects with rheumatoid arthritis, Crohn’s disease, obesity, and type 2 diabetes. Short-chain fatty acids (SCFAs), particularly acetate and butyrate, that are produced are thought to act as signaling molecules between the gut microbiome and host and may play a role in host metabolism (38). Unlike butyrate which seems to play a protective role in the gut microbiome (39), acetate is thought to interact with the host parasympathetic nervous system to modulate insulin secretion and may promote obesity (40, 41). Our finding that acetate production modules are consistently elevated across diseases supports prior work linking microbe-produced acetate to disease.

The integration of data from distinct diseases enables differentiation of disease-specific and disease-common indicators, which can clarify the etiology of specific diseases and advance their diagnosis. For example, rheumatoid arthritis cases carry an increased abundance of a methane production module (M00618) relative to controls. Increased abundance of methane-producing microorganisms was reported in patients with multiple sclerosis, an autoimmune disease that affects the central nervous system (42). These findings suggest that methane production by gut microbiota may associate with autoimmune conditions. Additionally, modules for degradation of glycosaminoglycans (GAGs) (M00076, M00077, M00078, and M00079) are uniquely elevated in subjects with Crohn’s disease. Increased degradation of GAGs in Crohn’s disease subjects has been reported previously (43) and may be caused by gut microbiota.

The observed indicators of disease also clarify the potential role of the microbiome in various diseases. By focusing on what the microbiome is capable of doing, rather than which taxa are present, and how this functional capacity associates with health, we can develop testable hypotheses about how the microbiome may mediate health and disease. For example, our work reveals robust associations between the functional composition of the gut microbiome and obesity. Among the indicators for obesity are modules for acetate production (M00377, M00579, and M00618). Recent research connects acetate production by gut microbiota to metabolic syndrome via interaction with the host parasympathetic nervous system to promote insulin secretion (40). These results are especially valuable in light of recent work that demonstrates an effect of the microbiome in metabolic diseases (44) but inconsistent (17) or weak (18) associations between the taxonomic composition of the gut microbiome and obesity. Notably, the overall functional diversity of the microbiome similarly manifests weak associations with obesity, but the aforementioned protein families robustly resolve the disease. Consequently, these specific indicators may serve as important leads in future studies of how the gut microbiome contributes to obesity and metabolic syndromes.

The random forest analysis demonstrates that the functional composition of the microbiome can aid in classifying disease status and may serve in disease diagnosis. However, the relatively large margin of error observed for some diseases or for classifying health versus disease in a general sense indicates that such diagnosis may be pertinent only for diseases with stronger microbiome signatures (i.e., Crohn’s disease or liver cirrhosis). As seen with colorectal cancer, the severity of the disease may also play a role in the potential for diagnostics.

Collectively, our analysis discerns how the gut microbiome’s functional capacity relates to host health. Through integration of data spanning multiple health states, we observe broad patterns of microbiome changes in disease that clarify how the gut microbiome contributes to health. For example, the metabolic modules that are commonly perturbed during disease may reflect mechanisms through which the gut microbiome interacts with physiology to promote health. Future studies should explicitly test whether the genes encoding these microbiome functions are actively expressed and critical to maintaining health. Moreover, disease associates with a personalized alteration in the functional composition in the microbiome, as indicated by our beta-diversity and beta-dispersion analyses. This result indicates that microbiome-based therapies may need to consider patient-specific parameters to ensure efficacy. Additionally, we uncover disease-specific indicators that not only serve as diagnostic leads but also clarify potential microbiome-mediated etiologies of disease. Future studies should similarly seek to test the effects of these microbiome functions on health. Expansion of metagenomic sampling across populations and health states is critical to advancing our understanding of how the functions encoded in the gut microbiome associate with disease, but improvements to existing analysis methodologies may be necessary to ensure that results are robust to technical considerations (e.g., the compositional nature of sequence data). Additionally, efforts to expand the functional characterization of microbial genes will enhance the sensitivity and specificity of imputed characterizations of microbiome functional capacity. Ultimately, integrative data analysis can expand our understanding of the role of the microbiome in maintaining health but requires more comprehensive patient data, standardized methodologies, and extended patient populations to maximize its utility.

## MATERIALS AND METHODS

### Data set.

Our analysis relied on public metagenomes, which we obtained from the NCBI SRA and identified using SRAdb (45). Specifically, we downloaded 10 Tbp of metagenomic sequence data from 1,979 subjects across 8 studies and 5 countries (see Table S1 in the supplemental material). Included in the data set are nondiseased controls as well as subjects with one or more of the following diseases: rheumatoid arthritis (11), colorectal cancer (6), liver cirrhosis (9), Crohn’s disease (10, 12), obesity (6–13), type 2 diabetes (6–8, 12, 13), and ulcerative colitis (10, 12) (Table S1). Sample covariates were obtained from the initial studies. While the publicly available metagenomes used in this study differed in their library sizes (number of reads), read length, and how they were quality controlled, a previous study found that the variation introduced by these factors is relatively small in contrast to biological variation between samples, indicating that these technical factors are unlikely to influence our results (27).

10.1128/mSystems.00332-18.4 TABLE S1 Data set and case/control count summary. Summary of data used in this analysis, including the total number of subjects for each disease examined in this analysis (see Materials and Methods for case/control selection criteria), country, and publications. Download Table S1, PDF file, 0.04 MB. Copyright © 2019 Armour et al. 2019 Armour et al. This content is distributed under the terms of the Creative Commons Attribution 4.0 International license.

10.1128/mSystems.00332-18.5 TABLE S2 Adonis *P* values and *r*^2^ values. *P* values and *r*^2^ values from Adonis on KEGG gene family abundance by host disease status. Boldface indicates significant values (*P* < 0.05) (vegan::adonis, 1,000 permutations). Variables are listed in the order in which they were included in Adonis. In rheumatoid arthritis and colorectal cancer, the disease levels were merged to a single case group. Download Table S2, PDF file, 0.05 MB. Copyright © 2019 Armour et al. 2019 Armour et al. This content is distributed under the terms of the Creative Commons Attribution 4.0 International license.

10.1128/mSystems.00332-18.6 TABLE S3 Results from compound Poisson linear regression with FDR correction. Summary of modeling results for each KEGG module in each disease. Publications associated with study abbreviations can be found in Table S1. Case and control mean columns are the average copy number across all case or control subjects. Download Table S3, CSV file, 0.4 MB. Copyright © 2019 Armour et al. 2019 Armour et al. This content is distributed under the terms of the Creative Commons Attribution 4.0 International license.

10.1128/mSystems.00332-18.7 TABLE S4 Summary of indicator discovery at different FDR thresholds. Counts of the number of indicator modules for each disease at several FDR thresholds. Download Table S4, PDF file, 0.04 MB. Copyright © 2019 Armour et al. 2019 Armour et al. This content is distributed under the terms of the Creative Commons Attribution 4.0 International license.

10.1128/mSystems.00332-18.8 TABLE S5 Summary of indicator overlap between diseases. Each indicator module appears once in the table with the diseases that it indicates. The definitions were obtained using KEGGREST. Also represented are the FDR values and the directionality of the change in abundance based on the slope estimate from the models. U indicates that the module is increased in abundance in the cases, and D indicates that the module is decreased in abundance in cases relative to controls. FDR values and change in cases are listed in the same order as in the disease column. Download Table S5, CSV file, 0.06 MB. Copyright © 2019 Armour et al. 2019 Armour et al. This content is distributed under the terms of the Creative Commons Attribution 4.0 International license.

### Data processing and annotation.

First, we pooled metagenomic reads across SRA run accessions (each representing a sequencing run) from the same SRA sample accession (each representing a biological sample). SRA samples were then functionally annotated using MetaQuery (46) to produce profiles of genomic copy number of KEGG Orthology Groups (KOs) in each metagenome. Briefly, MetaQuery uses Bowtie 2 (47) to align sample reads to the integrated gene catalog (IGC) of the human gut metagenome (8) to produce coverage estimates for each gene, which are functionally annotated by KOs. The coverage estimates are normalized by a set of 30 universal single-copy genes (48) to produce estimates of the genomic copy number of each KO in the microbiome. This statistic reflects the average number of gene copies per genome across all cells in a microbial community and is not influenced by differences in average genome size between samples (44). Thus, a universal single-copy gene will have an average genomic copy number close to 1.0. The classification rate of reads to the IGC ranged from 72 to 93% of reads with an average of 87% (Fig. S1). The classification of reads to KEGG annotated gene families ranged from 43 to 69% of reads, with an average of 55% (Fig. S1).

10.1128/mSystems.00332-18.2 FIG S1 Classification rate and number of reads classified to the integrated gene catalogue and KEGG database. (A) Box plots of the classification rates for cases (red) and controls (blue) in each disease to the IGC and KEGG database. Quantified as the number of reads assigned to a category in the database divided by the total number of reads in the sample. (B) Box plots of the number of reads classified for cases (red) and controls (blue) in each disease to the IGC and KEGG database. Horizontal dashed lines represent the mean number of reads across all the data (X for the IGC and Y for the KEGG database). Download FIG S1, EPS file, 0.2 MB. Copyright © 2019 Armour et al. 2019 Armour et al. This content is distributed under the terms of the Creative Commons Attribution 4.0 International license.

### Case and control population sampling.

For each disease, we identified a set of subjects that represented disease-afflicted individuals (i.e., cases) and a set of subjects that represented healthy individuals (i.e., controls) (Table S1). Specifically, we deemed subjects who were clearly determined to have the disease of interest and none of the other diseases in the available metadata to be case subjects (i.e., no comorbidity). Control populations alternatively consisted of individuals who were explicitly determined through clinical screening not to have the disease of interest, irrespective of the specific study from which the metagenome was generated. Designation of cases and controls relied on the metadata provided by the initial study. It is possible that a subject could have an undetected disease that was not screened for in a given study. Rheumatoid arthritis subjects who manifested low disease severity or remission were excluded from these analyses. Subjects with multiple SRA sample accessions were represented by only the first metagenome sample that the study authors generated. Ultimately, 1,473 samples passed these analytical filters and were included in the downstream analyses.

### Alpha- and beta-diversity.

The vegan package in R was used for alpha- and beta-diversity quantification. Specifically, the function specnumber (vegan::specnumber) assessed gene family richness, and a two-sided *t* test (stats::t.test) determined statistical significance between cases and controls within a disease. Beta-diversity was measured with Bray-Curtis dissimilarity (vegan::vegdist) and visualized with nonmetric multidimensional scaling (NMDS). Permutational multivariate analysis of variance (vegan::Adonis) calculated significant differences in beta-diversity. Beta-dispersion was quantified with betadisper (vegan::betadisper), and analysis of variance (ANOVA; stats::anova) determined significant differences.

### Identifying functions that stratify cases and control.

A regression-based approach modeled KEGG module abundances across populations to identify the functions that stratify cases and controls for each disease. To reduce dimensionality of the data, KOs were collapsed into modules and only modules with prevalence greater than 0.5 were tested. The model (cplm::cpglm) implements a Tweedie compound Poisson distribution with a degenerate distribution at the origin and a continuous distribution on the positive real line to appropriately model data where there are zeros but the values are otherwise continuous (29, 49). For each functional module, the normalized average genomic copy number was used as the response variable and disease status was used as the predictor. For disease phenotypes with data from multiple studies, the source study was also included as a covariate in the models to account for study effects. False discovery rate (FDR) correction (stat::p.adjust) was used to adjust for multiple tests and a cutoff of FDR < 0.2 was used to identify indicators for each disease.

### Identifying taxa that contribute to functional abundance.

To identify which taxa may drive the abundance of KEGG modules linked to disease, we used MetaPhlAn2 with default parameters to produce taxonomic annotations of each metagenome. Using these taxonomic abundance profiles, we correlated the relative abundance of each genus with the paired relative abundance of each indicator module across samples using an FDR-corrected Spearman test. We excluded samples where the abundance of the genus being examined was zero since we were interested only in assessing how the abundance of each module and genus correlates when the genus is present in the sample. Significant correlations were identified as those with FDR < 0.05 and |ρ| > 0.4. Correlation values are available in Table S6, and significant correlations are visualized in Fig. S2A to G.

10.1128/mSystems.00332-18.3 FIG S2 Heat maps of associations between genus and module abundance in each disease. The value in the heat map is the Spearman rho for all genus-module associations with FDR < 0.05 and |rho| > 0.4 for rheumatoid arthritis (A), colorectal cancer (B), liver cirrhosis (C), Crohn’s disease (D), obesity (E), type 2 diabetes (F), and ulcerative colitis (G). Module names on the *x* axis are not listed for liver cirrhosis (C), Crohn’s disease (D), obesity (E), and type 2 diabetes (F) since there are too many to print legibly. Exact values for each association can be obtained in Table S6. Download FIG S2, PDF file, 2.1 MB. Copyright © 2019 Armour et al. 2019 Armour et al. This content is distributed under the terms of the Creative Commons Attribution 4.0 International license.

10.1128/mSystems.00332-18.9 TABLE S6 Identification of genera that contribute to module abundance. Table of results from correlations between genus abundance and module abundance for each disease indicator module of each disease. KEGG module definition, Spearman’s rho, *P* value, and FDR from correlations between the abundance of each genus and indicator module for each disease. Download Table S6, CSV file, 0.3 MB. Copyright © 2019 Armour et al. 2019 Armour et al. This content is distributed under the terms of the Creative Commons Attribution 4.0 International license.

### Random forest classifier.

The R package randomForest (randomForest::randomForest) was used to quantify classification of subjects into the appropriate case or control categories for each disease. For the random forest by disease, the same populations of subjects were used for each disease as described above in the models. To quantify the ability to classify disease or nondisease, all data were gathered and subjects were given a label of diseased (1) or nondiseased (0) regardless of the disease. The subject metadata values (BMI, age, sex, and country) were added as additional variables. Subjects with NA (not available) values for any of the metadata variables were excluded (*n* = 137). We used the out-of-bag (OOB) error from the classifier and area under the curve (AUC) from receiver operating characteristic (ROC) curves (pROC::roc) of sensitivity and specificity to quantify the classification accuracy.

### Code availability.

The code to reproduce all analyses in the paper is available at https://github.com/courtneyarmour/human_metagenomes_analysis.

### Data availability.

Subject metadata and KEGG/MetaPhlAn2 annotated metagenomes are available at http://files.cgrb.oregonstate.edu/Sharpton_Lab/Papers/Armour_msystems_2019/.

## References

[1] Vuong HE, Yano JM, Fung TC, Hsiao EY 2017 The microbiome and host behavior. Annu Rev Neurosci 40:21–49. doi:10.1146/annurev-neuro-072116-031347. 28301775 PMC6661159

[2] Knight R, Callewaert C, Marotz C, Hyde ER, Debelius JW, McDonald D, Sogin ML 2017 The microbiome and human biology. Annu Rev Genomics Hum Genet 18:65–86. doi:10.1146/annurev-genom-083115-022438. 28375652

[3] Cho I, Blaser MJ 2012 The human microbiome: at the interface of health and disease. Nat Rev Genet 13:260–270. doi:10.1038/nrg3182. 22411464 PMC3418802

[4] Human Microbiome Project Consortium. 2012 Structure, function and diversity of the healthy human microbiome. Nature 486:207–214. doi:10.1038/nature11234. 22699609 PMC3564958

[5] Round JL, Mazmanian SK 2009 The gut microbiota shapes intestinal immune responses during health and disease. Nat Rev Immunol 9:313–323. doi:10.1038/nri2515. 19343057 PMC4095778

[6] Feng Q, Liang S, Jia H, Stadlmayr A, Tang L, Lan Z, Zhang D, Xia H, Xu X, Jie Z, Su L, Li X, Li X, Li J, Xiao L, Huber-Schönauer U, Niederseer D, Xu X, Al-Aama JY, Yang H, Wang J, Kristiansen K, Arumugam M, Tilg H, Datz C, Wang J 2015 Gut microbiome development along the colorectal adenoma-carcinoma sequence. Nat Commun 6:6528. doi:10.1038/ncomms7528. 25758642

[7] Karlsson FH, Tremaroli V, Nookaew I, Bergstrom G, Behre CJ, Fagerberg B, Nielsen J, Backhed F, Bergström G, Behre CJ, Fagerberg B, Nielsen J, Bäckhed F 2013 Gut metagenome in European women with normal, impaired and diabetic glucose control. Nature 498:99–103. doi:10.1038/nature12198. 23719380

[8] Li J, Jia H, Cai X, Zhong H, Feng Q, Sunagawa S, Arumugam M, Kultima JR, Prifti E, Nielsen T, Juncker AS, Manichanh C, Chen B, Zhang W, Levenez F, Wang J, Xu X, Xiao L, Liang S, Zhang D, Zhang Z, Chen W, Zhao H, Al-Aama JY, Edris S, Yang H, Wang J, Hansen T, Nielsen HB, Brunak S, Kristiansen K, Guarner F, Pedersen O, Doré J, Ehrlich SD, Bork P, Wang J 2014 An integrated catalog of reference genes in the human gut microbiome. Nat Biotechnol 32:834–841. doi:10.1038/nbt.2942. 24997786

[9] Qin N, Yang F, Li A, Prifti E, Chen YY, Shao L, Guo J, Le Chatelier E, Yao J, Wu L, Zhou J, Ni S, Liu L, Pons N, Batto JM, Kennedy SP, Leonard P, Yuan C, Ding W, Chen YY, Hu X, Zheng B, Qian G, Xu W, Ehrlich SD, Zheng S, Li L 2014 Alterations of the human gut microbiome in liver cirrhosis. Nature 513:59–64. doi:10.1038/nature13568. 25079328

[10] Nielsen HB, Almeida M, Juncker AS, Rasmussen S, Li J, Sunagawa S, Plichta DR, Gautier L, Pedersen AG, Le Chatelier E, Pelletier E, Bonde I, Nielsen T, Manichanh C, Arumugam M, Batto J-M, Quintanilha Dos Santos MB, Blom N, Borruel N, Burgdorf KS, Boumezbeur F, Casellas F, Doré J, Dworzynski P, Guarner F, Hansen T, Hildebrand F, Kaas RS, Kennedy S, Kristiansen K, Kultima JR, Léonard P, Levenez F, Lund O, Moumen B, Le Paslier D, Pons N, Pedersen O, Prifti E, Qin J, Raes J, Sørensen S, Tap J, Tims S, Ussery DW, Yamada T, MetaHIT Consortium, Renault P, Sicheritz-Ponten T, Bork P, Wang J, Brunak S, Ehrlich SD 2014 Identification and assembly of genomes and genetic elements in complex metagenomic samples without using reference genomes. Nat Biotechnol 32:822–832. doi:10.1038/nbt.2939. 24997787

[11] Zhang X, Zhang D, Jia H, Feng Q, Wang D, Liang D, Wu X, Li J, Tang L, Li Y, Lan Z, Chen B, Li Y, Zhong H, Xie H, Jie Z, Chen W, Tang S, Xu X, Wang X, Cai X, Liu S, Xia Y, Li J, Qiao X, Al-Aama JY, Chen H, Wang L, Wu Q-J, Zhang F, Zheng W, Li Y, Zhang M, Luo G, Xue W, Xiao L, Li J, Chen W, Xu X, Yin Y, Yang H, Wang J, Kristiansen K, Liu L, Li T, Huang Q, Li Y, Wang J 2015 The oral and gut microbiomes are perturbed in rheumatoid arthritis and partly normalized after treatment. Nat Med 21:895–905. doi:10.1038/nm.3914. 26214836

[12] Le Chatelier E, Nielsen T, Qin J, Prifti E, Hildebrand F, Falony G, Almeida M, Arumugam M, Batto J-M, Kennedy S, Leonard P, Li J, Burgdorf K, Grarup N, Jørgensen T, Brandslund I, Nielsen HB, Juncker AS, Bertalan M, Levenez F, Pons N, Rasmussen S, Sunagawa S, Tap J, Tims S, Zoetendal EG, Brunak S, Clement K, Dore J, Kleerebezem M, Kristiansen K, Renault P, Sicheritz-Ponten T, de Vos WM, Zucker J-D, Raes J, Hansen T, MetaHIT Consortium, Bork P, Wang J, Ehrlich SD, Pedersen O 2013 Richness of human gut microbiome correlates with metabolic markers. Nature 500:541–546. doi:10.1038/nature12506. 23985870

[13] Qin J, Li Y, Cai Z, Li S, Zhu J, Zhang F, Liang S, Zhang W, Guan Y, Shen D, Peng Y, Zhang D, Jie Z, Wu W, Qin Y, Xue W, Li J, Han L, Lu D, Wu P, Dai Y, Sun X, Li Z, Tang A, Zhong S, Li X, Chen W, Xu R, Wang M, Feng Q, Gong M, Yu J, Zhang Y, Zhang M, Hansen T, Sanchez G, Raes J, Falony G, Okuda S, Almeida M, LeChatelier E, Renault P, Pons N, Batto J-M, Zhang Z, Chen H, Yang R, Zheng W, Li S, Yang H, Wang J, Ehrlich SD, Nielsen R, Pedersen O, Kristiansen K, Wang J 2012 A metagenome-wide association study of gut microbiota in type 2 diabetes. Nature 490:55–60. doi:10.1038/nature11450. 23023125

[14] Haidich AB 2010 Meta-analysis in medical research. Hippokratia 14:29–37. 21487488 PMC3049418

[15] Duvallet C, Gibbons SM, Gurry T, Irizarry RA, Alm EJ 2017 Meta-analysis of gut microbiome studies identifies disease-specific and shared responses. Nat Commun 8:1784. doi:10.1038/s41467-017-01973-8. 29209090 PMC5716994

[16] Pasolli E, Truong DT, Malik F, Waldron L, Segata N 2016 Machine learning meta-analysis of large metagenomic datasets: tools and biological insights. PLoS Comput Biol 12:e1004977. doi:10.1371/journal.pcbi.1004977. 27400279 PMC4939962

[17] Finucane MM, Sharpton TJ, Laurent TJ, Pollard KS 2014 A taxonomic signature of obesity in the microbiome? Getting to the guts of the matter. PLoS One 9:e84689. doi:10.1371/journal.pone.0084689. 24416266 PMC3885756

[18] Sze MA, Schloss PD 2016 Looking for a signal in the noise: revisiting obesity and the microbiome. mBio 7:e01018-16. doi:10.1128/mBio.01018-16. 27555308 PMC4999546

[19] Hubbell SP 2006 Neutral theory and the evolution of ecological equivalence. Ecology 87:1387–1398. doi:10.1890/0012-9658(2006)87[1387:NTATEO]2.0.CO;2. 16869413

[20] Turnbaugh PJ, Ley RE, Hamady M, Fraser-Liggett CM, Knight R, Gordon JI 2007 The human microbiome project. Nature 449:804–810. doi:10.1038/nature06244. 17943116 PMC3709439

[21] Forslund K, Hildebrand F, Nielsen T, Falony G, Le Chatelier E, Sunagawa S, Prifti E, Vieira-Silva S, Gudmundsdottir V, Pedersen HK, Arumugam M, Kristiansen K, Voigt AY, Vestergaard H, Hercog R, Costea PI, Kultima JR, Li J, Jørgensen T, Levenez F, Dore J, MetaHIT Consortium, Nielsen HB, Brunak S, Raes J, Hansen T, Wang J, Ehrlich SD, Bork P, Pedersen O 2015 Disentangling type 2 diabetes and metformin treatment signatures in the human gut microbiota. Nature 528:262–266. doi:10.1038/nature15766. 26633628 PMC4681099

[22] Odamaki T, Kato K, Sugahara H, Hashikura N, Takahashi S, Xiao J-Z, Abe F, Osawa R 2016 Age-related changes in gut microbiota composition from newborn to centenarian: a cross-sectional study. BMC Microbiol 16:90. doi:10.1186/s12866-016-0708-5. 27220822 PMC4879732

[23] Yatsunenko T, Rey FE, Manary MJ, Trehan I, Dominguez-Bello MG, Contreras M, Magris M, Hidalgo G, Baldassano RN, Anokhin AP, Heath AC, Warner B, Reeder J, Kuczynski J, Caporaso JG, Lozupone CA, Lauber C, Clemente JC, Knights D, Knight R, Gordon JI 2012 Human gut microbiome viewed across age and geography. Nature 486:222–227. doi:10.1038/nature11053. 22699611 PMC3376388

[24] Yun Y, Kim H-N, Kim SE, Heo SG, Chang Y, Ryu S, Shin H, Kim H-L 2017 Comparative analysis of gut microbiota associated with body mass index in a large Korean cohort. BMC Microbiol 17:151. doi:10.1186/s12866-017-1052-0. 28676106 PMC5497371

[25] Haro C, Rangel-Zúñiga OA, Alcalá-Díaz JF, Gómez-Delgado F, Pérez-Martínez P, Delgado-Lista J, Quintana-Navarro GM, Landa BB, Navas-Cortés JA, Tena-Sempere M, Clemente JC, López-Miranda J, Pérez-Jiménez F, Camargo A 2016 Intestinal microbiota is influenced by gender and body mass index. PLoS One 11:e0154090. doi:10.1371/journal.pone.0154090. 27228093 PMC4881937

[26] Gibbons SM, Duvallet C, Alm EJ 2018 Correcting for batch effects in case-control microbiome studies. PLoS Comput Biol 14:e1006102. doi:10.1371/journal.pcbi.1006102. 29684016 PMC5940237

[27] Nayfach S, Pollard KS 2016 Toward accurate and quantitative comparative metagenomics. Cell 166:1103–1116. doi:10.1016/j.cell.2016.08.007. 27565341 PMC5080976

[28] Zaneveld JR, McMinds R, Thurber RV 2017 Stress and stability: applying the Anna Karenina principle to animal microbiomes. Nat Microbiol 2:17121. doi:10.1038/nmicrobiol.2017.121. 28836573

[29] Sharpton T, Lyalina S, Luong J, Pham J, Deal EM, Armour C, Gaulke C, Sanjabi S, Pollard KS 2017 Development of inflammatory bowel disease is linked to a longitudinal restructuring of the gut metagenome in mice. mSystems 2:e00036-17. doi:10.1128/mSystems.00036-17. 28904997 PMC5585689

[30] Dasgupta N, Kapur V, Singh KK, Das TK, Sachdeva S, Jyothisri K, Tyagi JS 2000 Characterization of a two-component system, devR-devS, of Mycobacterium tuberculosis. Tuber Lung Dis 80:141–159. doi:10.1054/tuld.2000.0240. 10970762

[31] Truong DT, Franzosa EA, Tickle TL, Scholz M, Weingart G, Pasolli E, Tett A, Huttenhower C, Segata N 2015 MetaPhlAn2 for enhanced metagenomic taxonomic profiling. Nat Methods 12:902–903. doi:10.1038/nmeth.3589. 26418763

[32] Zeller G, Tap J, Voigt AY, Sunagawa S, Kultima JR, Costea PI, Amiot A, Bohm J, Brunetti F, Habermann N, Hercog R, Koch M, Luciani A, Mende DR, Schneider MA, Schrotz-King P, Tournigand C, Tran Van Nhieu J, Yamada T, Zimmermann J, Benes V, Kloor M, Ulrich CM, von Knebel Doeberitz M, Sobhani I, Bork P 2014 Potential of fecal microbiota for early-stage detection of colorectal cancer. Mol Syst Biol 10:766. doi:10.15252/msb.20145645. 25432777 PMC4299606

[33] Wirbel J, Pyl PT, Kartal E, Zych K, Kashani A, Milanese A, Fleck JS, Voigt AY, Palleja A, Ponnudurai R, Sunagawa S, Coelho LP, Schrotz-King P, Vogtmann E, Habermann N, Niméus E, Thomas AM, Manghi P, Gandini S, Serrano D, Mizutani S, Shiroma H, Shiba S, Shibata T, Yachida S, Yamada T, Waldron L, Naccarati A, Segata N, Sinha R, Ulrich CM, Brenner H, Arumugam M, Bork P, Zeller G 2019 Meta-analysis of fecal metagenomes reveals global microbial signatures that are specific for colorectal cancer. Nat Med 25:679–689. doi:10.1038/s41591-019-0406-6. 30936547 PMC7984229

[34] Thomas AM, Manghi P, Asnicar F, Pasolli E, Armanini F, Zolfo M, Beghini F, Manara S, Karcher N, Pozzi C, Gandini S, Serrano D, Tarallo S, Francavilla A, Gallo G, Trompetto M, Ferrero G, Mizutani S, Shiroma H, Shiba S, Shibata T, Yachida S, Yamada T, Wirbel J, Schrotz-King P, Ulrich CM, Brenner H, Arumugam M, Bork P, Zeller G, Cordero F, Dias-Neto E, Setubal JC, Tett A, Pardini B, Rescigno M, Waldron L, Naccarati A, Segata N 2019 Metagenomic analysis of colorectal cancer datasets identifies cross-cohort microbial diagnostic signatures and a link with choline degradation. Nat Med 25:667–678. doi:10.1038/s41591-019-0405-7. 30936548 PMC9533319

[35] Mosca A, Leclerc M, Hugot JP 2016 Gut microbiota diversity and human diseases: should we reintroduce key predators in our ecosystem? Front Microbiol 7:455. doi:10.3389/fmicb.2016.00455. 27065999 PMC4815357

[36] Panek M, Čipčić Paljetak H, Barešić A, Perić M, Matijašić M, Lojkić I, Vranešić Bender D, Krznarić Ž, Verbanac D 2018 Methodology challenges in studying human gut microbiota—effects of collection, storage, DNA extraction and next generation sequencing technologies. Sci Rep 8:5143. doi:10.1038/s41598-018-23296-4. 29572539 PMC5865204

[37] Skaar EP 2010 The battle for iron between bacterial pathogens and their vertebrate hosts. PLoS Pathog 6:e1000949. doi:10.1371/journal.ppat.1000949. 20711357 PMC2920840

[38] Morrison DJ, Preston T 2016 Formation of short chain fatty acids by the gut microbiota and their impact on human metabolism. Gut Microbes 7:189–200. doi:10.1080/19490976.2015.1134082. 26963409 PMC4939913

[39] Geirnaert A, Calatayud M, Grootaert C, Laukens D, Devriese S, Smagghe G, De Vos M, Boon N, Van de Wiele T 2017 Butyrate-producing bacteria supplemented in vitro to Crohn’s disease patient microbiota increased butyrate production and enhanced intestinal epithelial barrier integrity. Sci Rep 7:11450. doi:10.1038/s41598-017-11734-8. 28904372 PMC5597586

[40] Perry RJ, Peng L, Barry NA, Cline GW, Zhang D, Cardone RL, Petersen KF, Kibbey RG, Goodman AL, Shulman GI 2016 Acetate mediates a microbiome–brain–β-cell axis to promote metabolic syndrome. Nature 534:213–217. doi:10.1038/nature18309. 27279214 PMC4922538

[41] Trent CM, Blaser MJ 2016 Microbially produced acetate: a “missing link” in understanding obesity? Cell Metab 24:9–10. doi:10.1016/j.cmet.2016.06.023. 27411005

[42] Jangi S, Gandhi R, Cox LM, Li N, Von Glehn F, Yan R, Patel B, Mazzola MA, Liu S, Glanz BL, Cook S, Tankou S, Stuart F, Melo K, Nejad P, Smith K, Topçuolu BD, Holden J, Kivisäkk P, Chitnis T, De Jager PL, Quintana FJ, Gerber GK, Bry L, Weiner HL 2016 Alterations of the human gut microbiome in multiple sclerosis. Nat Commun 7:12015. doi:10.1038/ncomms12015. 27352007 PMC4931233

[43] Murch SH, MacDonald TT, Walker-Smith JA, Lionetti P, Levin M, Klein NJ 1993 Disruption of sulphated glycosaminoglycans in intestinal inflammation. Lancet 341:711–714. doi:10.1016/0140-6736(93)90485-Y. 8095623

[44] Turnbaugh PJ, Ley RE, Mahowald MA, Magrini V, Mardis ER, Gordon JI 2006 An obesity-associated gut microbiome with increased capacity for energy harvest. Nature 444:1027–1031. doi:10.1038/nature05414. 17183312

[45] Zhu Y, Stephens RM, Meltzer PS, Davis SR 2013 SRAdb: query and use public next-generation sequencing data from within R. BMC Bioinformatics 14:19. doi:10.1186/1471-2105-14-19. 23323543 PMC3560148

[46] Nayfach S, Fischbach MA, Pollard KS 2015 MetaQuery: a web server for rapid annotation and quantitative analysis of specific genes in the human gut microbiome. Bioinformatics 31:3368–3370. doi:10.1093/bioinformatics/btv382. 26104745 PMC4595903

[47] Langmead B, Salzberg SL 2012 Fast gapped-read alignment with Bowtie 2. Nat Methods 9:357–359. doi:10.1038/nmeth.1923. 22388286 PMC3322381

[48] Nayfach S, Pollard KS 2015 Average genome size estimation improves comparative metagenomics and sheds light on the functional ecology of the human microbiome. Genome Biol 16:51. doi:10.1186/s13059-015-0611-7. 25853934 PMC4389708

[49] Zhang Y 2013 Likelihood-based and Bayesian methods for Tweedie compound Poisson linear mixed models. Stat Comput 23:743–757. doi:10.1007/s11222-012-9343-7.

