# Supplementary material for: A Metagenomic Meta-analysis Reveals Functional Signatures of Health and Disease in the Human Gut Microbiome
Source: mSystems. 2019 May 14;4(4):e00332-18. doi: 10.1128/mSystems.00332-18 (PMC6517693; doi:10.1128/mSystems.00332-18)
Supplement: TEXT S1 [file mSystems.00332-18-s0001.pdf]

Supplemental Text 1

Quantifying contribution of DNA sequencing method to variance in gut microbiome module abundance

An important consideration of microbiome analyses is the numerous factors that can introduce variation into the composition of the gut microbiome and confound analysis. This includes factors such as DNA extraction (5), library size, read length, and quality control(6). A prior analysis with a subset of this data found that the variation introduced by library size, read length, and quality control parameters is relatively small in contrast to biological variation between samples, indicating that these technical factors are unlikely to influence our results. Because DNA extraction method can by a major confounder, we explored how it affects our data. We identified the DNA extraction protocol for several studies and quantified its contribution to variation in beta diversity. Unfortunately, most of the studies in this analysis do not detail the protocol used for DNA extraction. For example, the rheumatoid arthritis study (1) states DNA was extracted “according to the protocol for fecal samples” with no reference or additional information and the colorectal cancer (2) study does not specify a DNA extraction protocol. Additionally, the type II diabetes study from Sweden (3) references another paper for their DNA extraction methods (4). However, the paper they reference is a comparison of DNA extraction methodology, so it is unclear which method they are referencing. The four remaining studies used one of two different DNA extraction approaches: phenol chloroform- and guanidine thiocyanate-based protocols. The guanidine thiocyanate method was used by the liver cirrhosis study from China (7) and the MetaHIT studies (8, 9). A separate study from China (10) used a phenol chloroform

DNA extraction protocol. Since these datasets include subjects with several different diseases, we selected just the non-diseased controls for comparison to reduce confounding variables related to health status. We quantified Bray-Curtis dissimilarity of KEGG protein family abundance profiles and conducted a PERMANOVA analysis to quantify the contribution of DNA extraction protocol to beta diversity. Overall, we find that protocol explains very little variation (supplemental text figure 1, PERMANOVA, $p < 0.01$, $R^2 = 0.036$) relative to the variation explained by geographic region (supplemental text figure 1A, PERMANOVA, $p < 0.01$, $R^2 = 0.143$) and study (supplemental text figure 1B, PERMANOVA, $p < 0.01$, $R^2 = 0.146$). When we applied a multivariate equation to PERMANOVA to understand the variation in community beta diversity, we found that including a variable for study made the DNA protocol term non-significant. A similar model that included variables for geographic region and protocol found that protocol is significant but explains very little between-sample variance (PERMANOVA $p < 0.01$, $R^2 = 0.003$). We conclude that DNA extraction protocol accounts for a relatively unsubstantial source of variation in our analysis and that geographic region and study contribute much more of the observed variation.

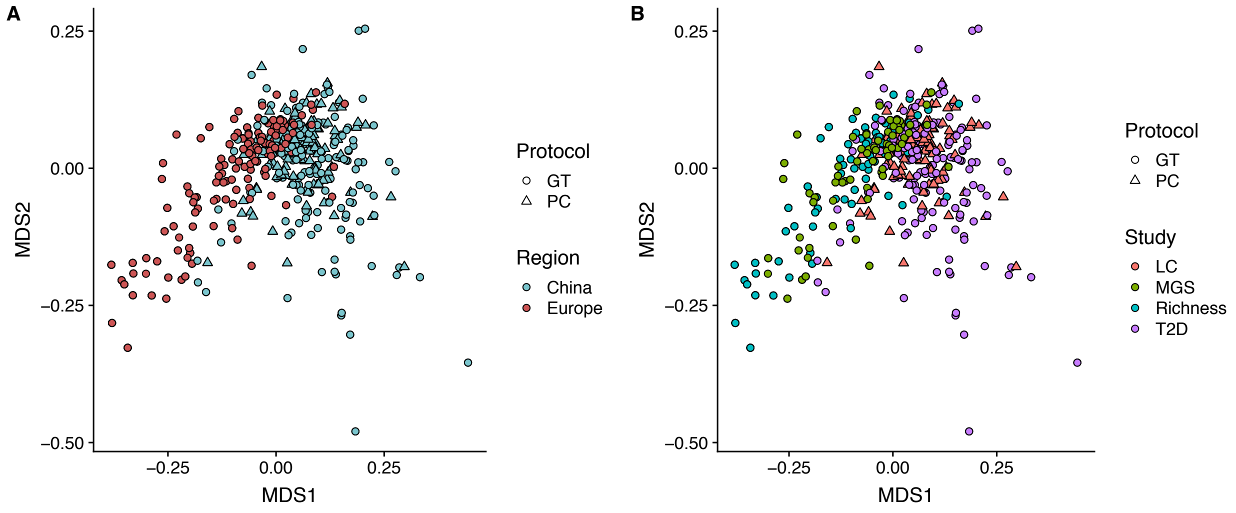

Supplemental Text Figure 1: Non-metric multidimensional scaling of Bray-Curtis dissimilarity on KEGG protein family abundance colored by A) geographic region and B) study. The references for the studies can be found in supplemental table 1. The samples cluster more by geographic region or study than buy DNA extraction protocol.

Comparison of regression results of the merged datasets to individual datasets

In the discovery of disease indicator modules, we integrated multiple datasets to improve sample size and power. Integration of data allows us to identify microbiome indicators that are robust across populations. However, it is unclear how robust these indicators are to individual populations. We ran CPLM regression models (as described in methods) to model module abundance as a function of health status for each individual study of obesity and type II diabetes and compared the resulting p-values to those from the regression analysis on the merged dataset. We found that the results for the largest individual study correlated with the results for the merged data (supplemental text figure 2). There was no significant association between the full dataset and smaller studies. These results could indicate microbiome associations are not robust across studies, but they could also result from some studies being insufficiently powered to resolve the association. In support of the latter, many of the p-values that are small in the full dataset have a range of p-values (0 to 1) analyses of individual studies, suggesting that the small studies may be underpowered. For each of the individual studies, there is overlap in significant modules, however the amount of overlap varies by study (supplemental text figure 2)."

A) Type II Diabetes

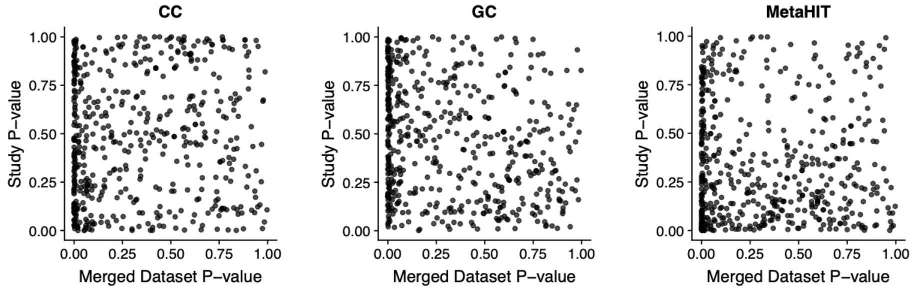

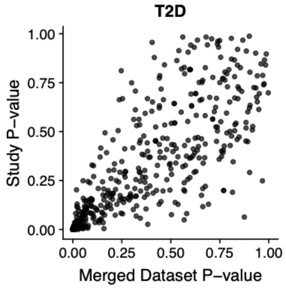

Type II Diabetes Sample Sizes and P-value Correlations				
Study Country	Control Count	Case Count	Spearman's rho	Count p < 0.05 (# p<0.05 in full dataset)
CC Austria	35	6	0.10	46 (11)
GC Sweden	36	24	-0.21	27 (5)
MetaHIT (Richness/ IGC)	112	17	0.01	86 (34)
T2D china	183	156	0.89	154 (135)

B) Obesity

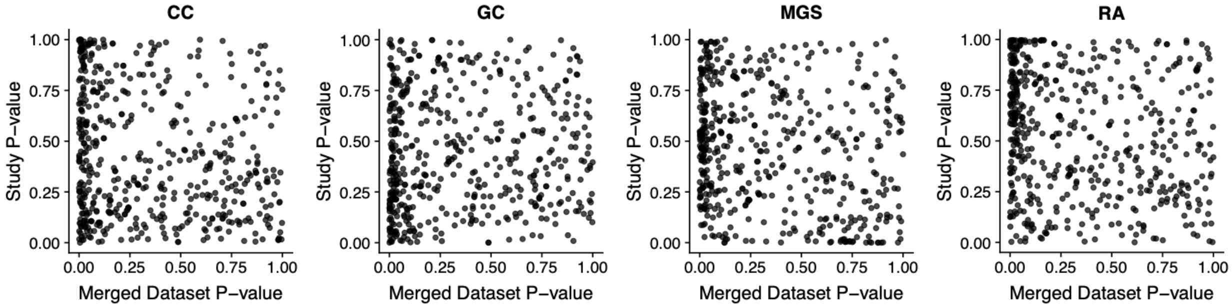

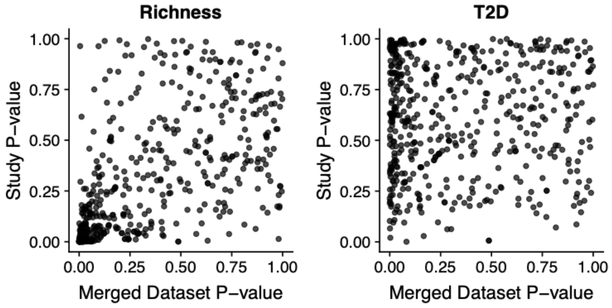

Obesity Sample Sizes and P-value Correlations				
Study Country	Control Count	Case Count	Spearman's rho	Count p < 0.05 (# p<0.05 in full dataset)
CC Austria	35	15	-0.18	34 (7)
GC Sweden	76	16	0.09	30 (10)
MGS Spain	66	5	-0.20	49 (1)
RA China	88	4	-0.22	23 (2)
Richness Denmark	122	169	0.70	147 (98)
T2D china	183	2	0.01	5 (1)

Supplemental Text Figure 2: Scatterplots of p-values obtained from CPLM regression where the results from the merged dataset are represented on the x-axis and the results from each individual study are represented on the y-axis with a separate plot for each study. Tables quantify sample sizes, Spearman's rho, the number of significant indicator modules, and how many of the significant indicator modules overlap with the indicator modules from the full dataset. The individual studies with larger sample sizes tend to correlate with the merged dataset, while studies with smaller samples sizes do not.

References

1. Costea PI, Zeller G, Sunagawa S, Pelletier E, Alberti A, Levenez F, Tramontano M, Driessen M, Hercog R, Jung F-E, Kultima JR, Hayward MR, Coelho LP, Allen-Vercoe E, Bertrand L, Blaut M, Brown JRM, Carton T, Cools-Portier S, Daigneault M, Derrien M, Druesne A, de Vos WM, Finlay BB, Flint HJ, Guarner F, Hattori M, Heilig H, Luna RA, van Hylckama Vlieg J, Junick J, Klymiuk I, Langella P, Le Chatelier E, Mai V, Manichanh C, Martin JC, Mery C, Morita H, O'Toole PW, Orvain C, Patil KR, Penders J, Persson S, Pons N, Popova M, Salonen A, Saulnier D, Scott KP, Singh B, Slezak K, Veiga P, Versalovic J, Zhao L, Zoetendal EG, Ehrlich SD, Dore J, Bork P. 2017. Towards standards for human fecal sample processing in metagenomic studies. Nat Biotechnol.
2. Nayfach S, Pollard KS. 2016. Toward Accurate and Quantitative Comparative Metagenomics. Cell 166:1103–1116.
3. Zhang X, Zhang D, Jia H, Feng Q, Wang D, Liang D, Wu X, Li J, Tang L, Li Y, Lan Z, Chen B, Li Y, Zhong H, Xie H, Jie Z, Chen W, Tang S, Xu X, Wang X, Cai X, Liu S, Xia Y, Li J, Qiao X, Al-Aama JY, Chen H, Wang L, Wu Q-J, Zhang F, Zheng W, Li Y, Zhang M, Luo G, Xue W, Xiao L, Li J, Chen W, Xu X, Yin Y, Yang H, Wang J, Kristiansen K, Liu L, Li T, Huang Q, Li Y, Wang J. 2015. The oral and gut microbiomes are perturbed in rheumatoid arthritis and partly normalized after treatment. Nat Med 21:895–905.
4. Feng Q, Liang S, Jia H, Stadlmayr A, Tang L, Lan Z, Zhang D, Xia H, Xu X, Jie Z, Su L, Li X, Li X, Li J, Xiao L, Huber-Schönauer U, Niederseer D, Xu X, Al-Aama JY, Yang H, Wang J, Kristiansen K, Arumugam M, Tilg H, Datz C, Wang J. 2015.

Gut microbiome development along the colorectal adenoma-carcinoma sequence. Nat Commun 6:6528.

5. Karlsson FH, Tremaroli V, Nookaew I, Bergstrom G, Behre CJ, Fagerberg B, Nielsen J, Backhed F, Bergström G, Behre CJ, Fagerberg B, Nielsen J, Bäckhed F. 2013. Gut metagenome in European women with normal, impaired and diabetic glucose control. Nature 498:99–103.

6. Salonen A, de Vos WM, Palva A. 2010. Gastrointestinal microbiota in irritable bowel syndrome: present state and perspectives. Microbiology 156:3205–3215.

7. Qin N, Yang F, Li A, Prifti E, Chen YY, Shao L, Guo J, Le Chatelier E, Yao J, Wu L, Zhou J, Ni S, Liu L, Pons N, Batto JM, Kennedy SP, Leonard P, Yuan C, Ding W, Chen YY, Hu X, Zheng B, Qian G, Xu W, Ehrlich SD, Zheng S, Li L. 2014. Alterations of the human gut microbiome in liver cirrhosis. Nature 513:59–64.

8. Nielsen HB, Almeida M, Juncker AS, Rasmussen S, Li J, Sunagawa S, Plichta DR, Gautier L, Pedersen AG, Le Chatelier E, Pelletier E, Bonde I, Nielsen T, Manichanh C, Arumugam M, Batto J-M, Quintanilha Dos Santos MB, Blom N, Borruel N, Burgdorf KS, Boumezbeur F, Casellas F, Doré J, Dworzynski P, Guarner F, Hansen T, Hildebrand F, Kaas RS, Kennedy S, Kristiansen K, Kultima JR, Léonard P, Levenez F, Lund O, Moumen B, Le Paslier D, Pons N, Pedersen O, Prifti E, Qin J, Raes J, Sørensen S, Tap J, Tims S, Ussery DW, Yamada T, MetaHIT Consortium, Renault P, Sicheritz-Ponten T, Bork P, Wang J, Brunak S, Ehrlich SD. 2014. Identification and assembly of genomes and genetic elements in complex metagenomic samples without using reference genomes. Nat Biotechnol 32:822–832.

9. Le Chatelier E, Nielsen T, Qin J, Prifti E, Hildebrand F, Falony G, Almeida M, Arumugam M, Batto J-M, Kennedy S, Leonard P, Li J, Burgdorf K, Grarup N, Jørgensen T, Brandslund I, Nielsen HB, Juncker AS, Bertalan M, Levenez F, Pons N, Rasmussen S, Sunagawa S, Tap J, Tims S, Zoetendal EG, Brunak S, Clement K, Dore J, Kleerebezem M, Kristiansen K, Renault P, Sicheritz-Ponten T, de Vos WM, Zucker J-DD, Raes J, Hansen T, MetaHIT Consortium, Bork P, Wang J, Ehrlich SD, Pedersen O. 2013. Richness of human gut microbiome correlates with metabolic markers. Nature 500:541–546.

10. Qin J, Li Y, Cai Z, Li S, Zhu J, Zhang F, Liang S, Zhang W, Guan Y, Shen D, Peng Y, Zhang D, Jie Z, Wu W, Qin Y, Xue W, Li J, Han L, Lu D, Wu P, Dai Y, Sun X, Li Z, Tang A, Zhong S, Li X, Chen W, Xu R, Wang M, Feng Q, Gong M, Yu J, Zhang Y, Zhang M, Hansen T, Sanchez G, Raes J, Falony G, Okuda S, Almeida M, LeChatelier E, Renault P, Pons N, Batto J-M, Zhang Z, Chen H, Yang R, Zheng W, Li S, Yang H, Ehrlich SD, Nielsen R, Pedersen O, Kristiansen K, Wang J. 2012. A metagenome-wide association study of gut microbiota in type 2 diabetes. Nature 490:55–60.
