# Supplementary material for: A Metagenomic Meta-analysis Reveals Functional Signatures of Health and Disease in the Human Gut Microbiome
Source: mSystems. 2019 May 14;4(4):e00332-18. doi: 10.1128/mSystems.00332-18 (PMC6517693; doi:10.1128/mSystems.00332-18)
Supplement: TABLE S1 [file mSystems.00332-18-st001.pdf]

Table S1

Disease	Disease Level	Cases	Controls	Studies	Host Countries	PubMed ID	Citation
Rheumatoid Arthritis	Moderate	45	93	RA	China	26214836	Zhang 2015
	High	70					
Colorectal Cancer	Advanced adenoma	28	35	CC	Austria	25758642	Feng 2015
	Carcinoma	36					
Liver Cirrhosis	-	120	114	LC	China	25079328	Qin 2014
Crohn's Disease	-	12	132	Richness	Denmark	23985870	Le Chatelier 2013
				MGS	Spain, Denmark	24997787	Nielsen 2014
Obesity	-	211	684	GC	Sweden	23719380	Karlsson 2013
				CC	Austria	25758642	Feng 2015
				RA	China	26214836	Zhang 2015
				T2D	China	23023125	Qin 2012
				LC	China	25079328	Qin 2014
				Richness	Denmark	23985870	Le Chatelier 2013
				MGS	Spain, Denmark	24997787	Nielsen 2014
Type II Diabetes	-	204	366	GC	Sweden	23719380	Karlsson 2013
				CC	Austria	25758642	Feng 2015
				T2D	China	23023125	Qin 2012
				Richness	Denmark	23985870	Le Chatelier 2013
				IGC	Spain, Denmark	24997786	Li 2014
Ulcerative colitis	-	58	132	Richness	Denmark	23985870	Le Chatelier 2013
				MGS	Spain, Denmark	24997787	Nielsen 2014
