# Supplementary material for: A Metagenomic Meta-analysis Reveals Functional Signatures of Health and Disease in the Human Gut Microbiome
Source: mSystems. 2019 May 14;4(4):e00332-18. doi: 10.1128/mSystems.00332-18 (PMC6517693; doi:10.1128/mSystems.00332-18)
Supplement: TABLE S2 [file mSystems.00332-18-st002.pdf]

Table S2

Disease	Variable	Adonis p-value	Adonis R-squared
Rheumatoid arthritis	status	0.223776	0.64%
	age	0.693307	0.33%
	bmi	0.324675	0.51%
	sex	0.487512	0.43%
Colorectal cancer	status	**0.047952**	**2.06%**
	age	**0.007992**	**3.26%**
	bmi	**0.024975**	**2.57%**
	sex	0.697303	0.60%
Liver Cirrhosis	status	**0.000999**	**3.33%**
	age	0.473526	0.38%
	bmi	0.134865	0.64%
	sex	**0.000999**	**1.64%**
Crohn's Disease	status	**0.000999**	**10.29%**
	age	**0.026973**	**1.78%**
	bmi	0.312687	0.73%
	sex	0.425574	0.59%
Obesity	status	**0.000999**	**1.22%**
	age	**0.000999**	**8.46%**
	bmi	0.109890	0.16%
	sex	**0.001998**	**0.63%**
	study	**0.000999**	**18.14%**
Type II Diabetes	status	**0.000999**	**1.68%**
	age	**0.000999**	**9.26%**
	bmi	0.353646	0.15%
	sex	0.072927	0.30%
	study	**0.000999**	**14.92%**
Ulcerative Colitis	status	**0.009990**	**1.45%**
	age	**0.015984**	**1.54%**
	bmi	0.779221	0.30%
	sex	0.511489	0.44%
