# Supplementary material for: A Metagenomic Meta-analysis Reveals Functional Signatures of Health and Disease in the Human Gut Microbiome
Source: mSystems. 2019 May 14;4(4):e00332-18. doi: 10.1128/mSystems.00332-18 (PMC6517693; doi:10.1128/mSystems.00332-18)
Supplement: TABLE S4 [file mSystems.00332-18-st004.pdf]

Table S4

	Level	FDR Range		Count below FDR threshold			
		Min FDR	Max FDR	0.05	0.1	0.15	0.2
**Rheumatoid Arthritis**	KO	0.00065	1.00000	19	57	121	158
	module	0.00256	0.99987	9	16	18	20
**Colorectal Cancer**	KO	0.00012	1.00000	119	248	416	641
	module	0.00186	1.00000	5	10	16	51
**Liver Cirrhosis**	KO	0.00000	1.00000	2872	3245	3576	3865
	module	0.00000	0.98609	252	285	306	333
**Crohn's Disease**	KO	0.00000	1.00000	3221	3525	3729	3902
	module	0.00000	1.00000	295	319	342	349
**Obesity**	KO	0.00000	0.99956	104	361	799	1338
	module	0.00024	0.99985	15	29	116	144
**Type II Diabetes**	KO	0.00000	1.00000	958	1277	1506	1719
	module	0.00001	0.99719	101	127	141	170
**Ulcerative Colitis**	KO	0.00340	1.00000	314	335	388	435
	module	0.00995	0.99948	11	11	12	13
