# Supplementary figures and images for: A Metagenomic Meta-analysis Reveals Functional Signatures of Health and Disease in the Human Gut Microbiome
Source: mSystems. 2019 May 14;4(4):e00332-18. doi: 10.1128/mSystems.00332-18 (PMC6517693; doi:10.1128/mSystems.00332-18)

Figure S2 A

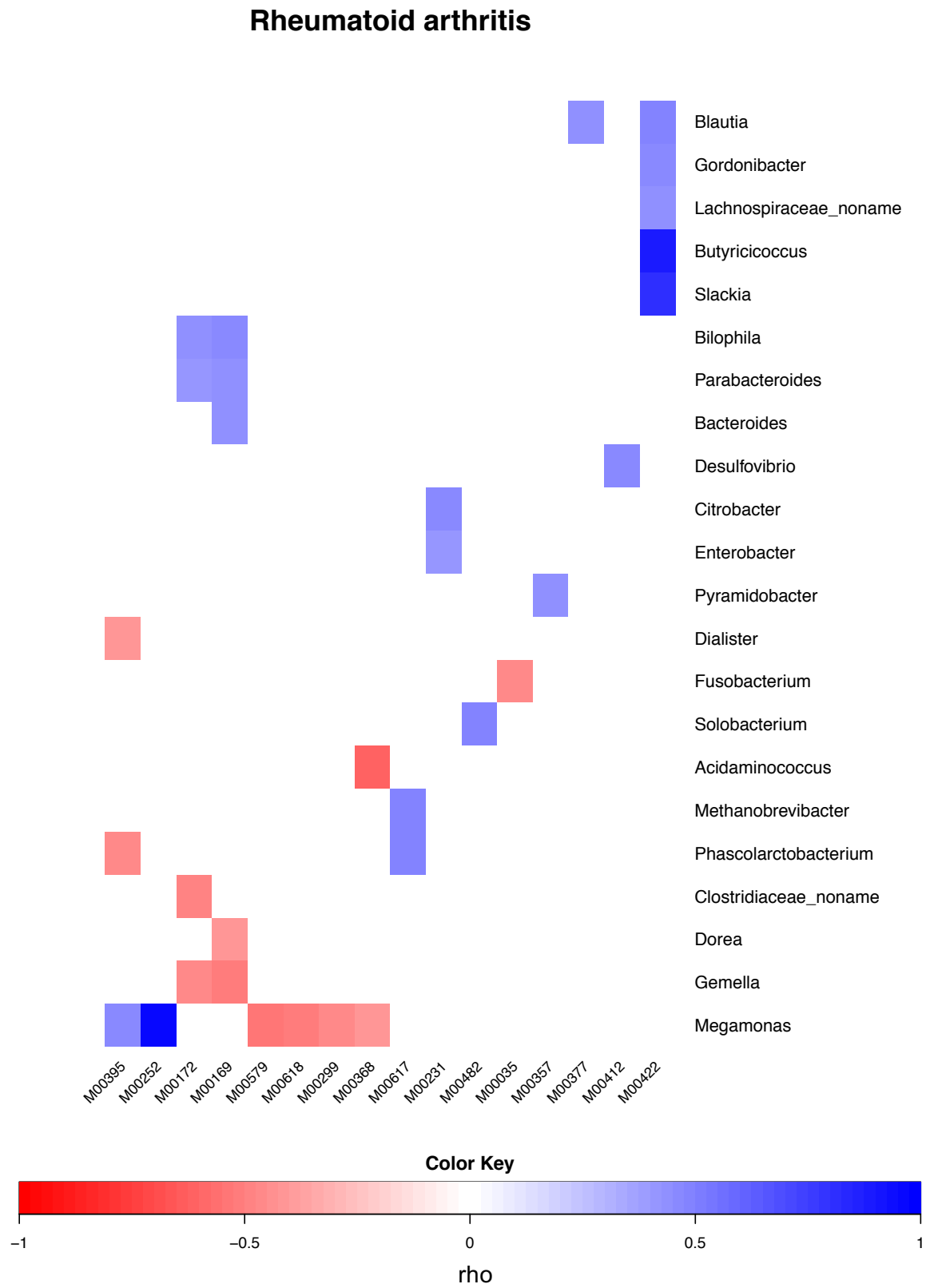

Figure S2 B

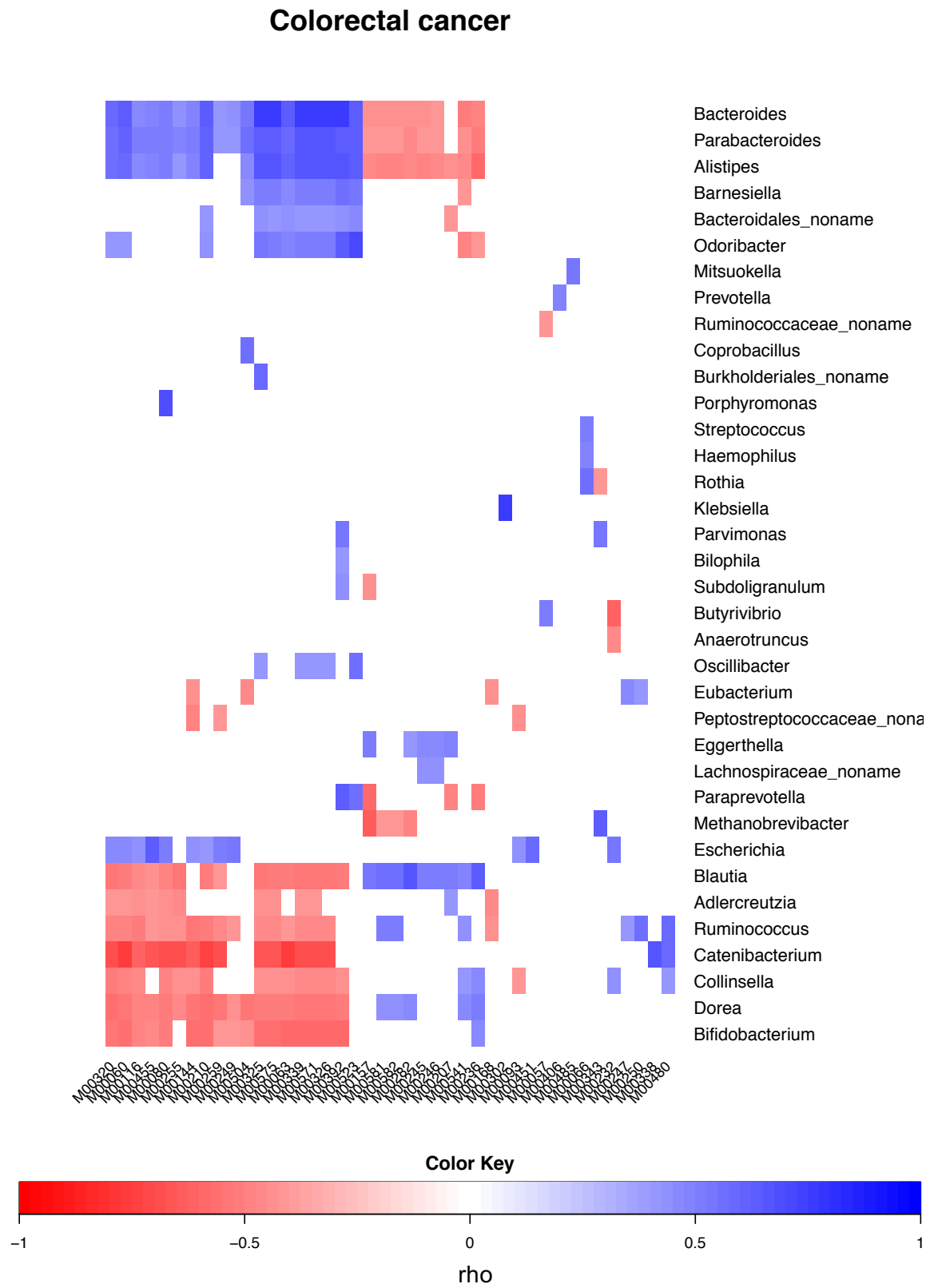

Figure S2 C

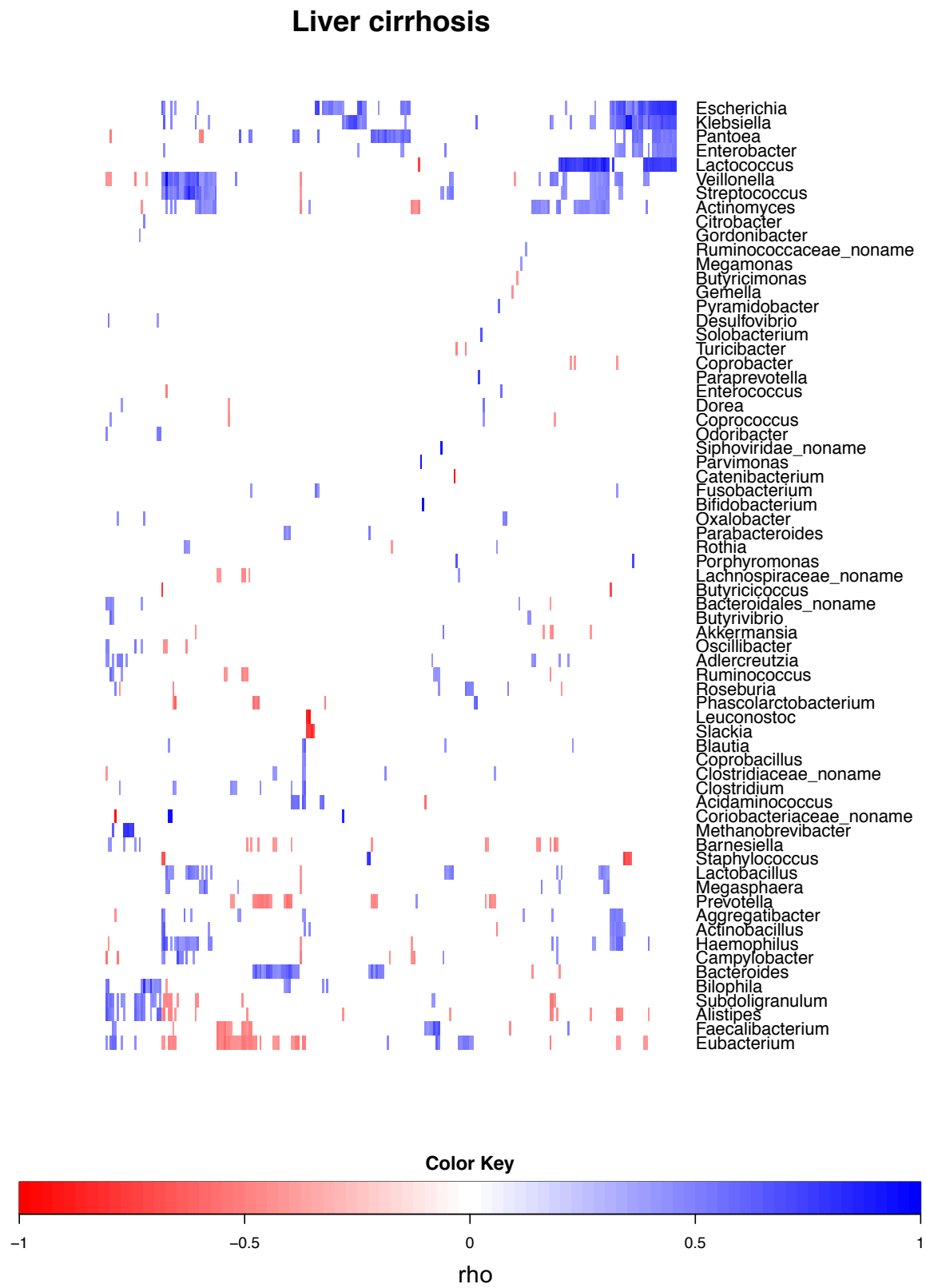

# Figure S2 D

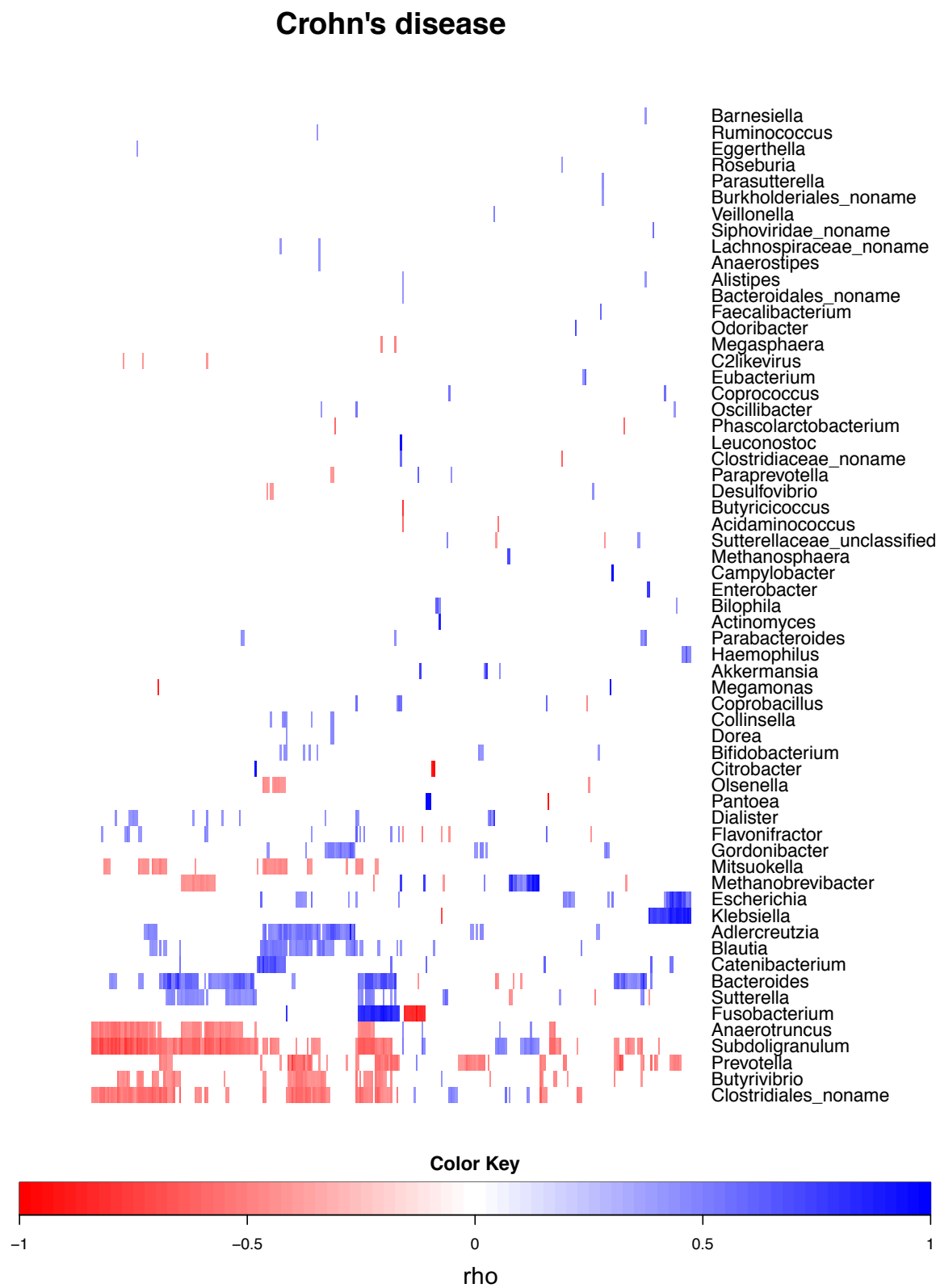

Figure S2 E

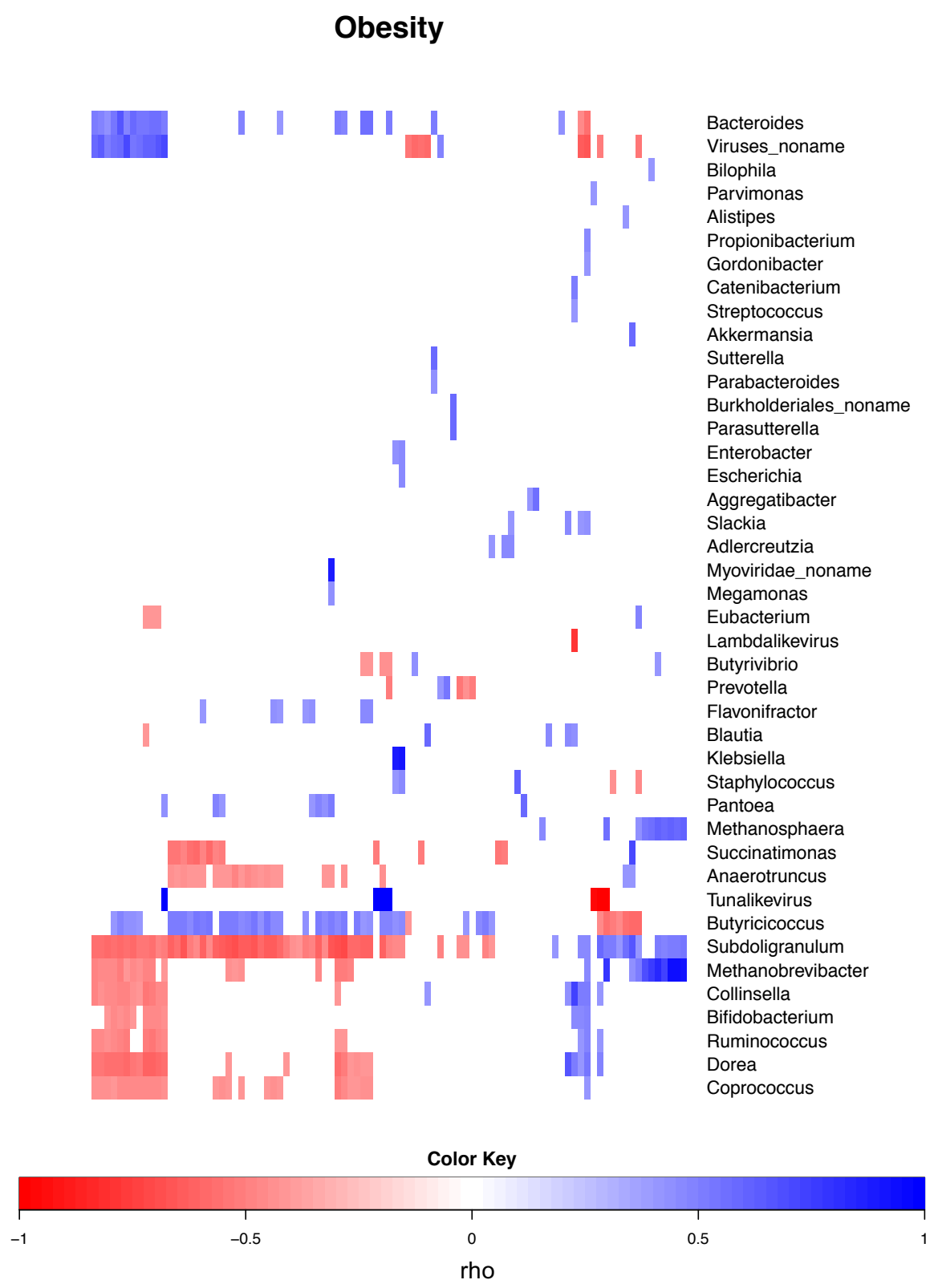

Figure S2 F

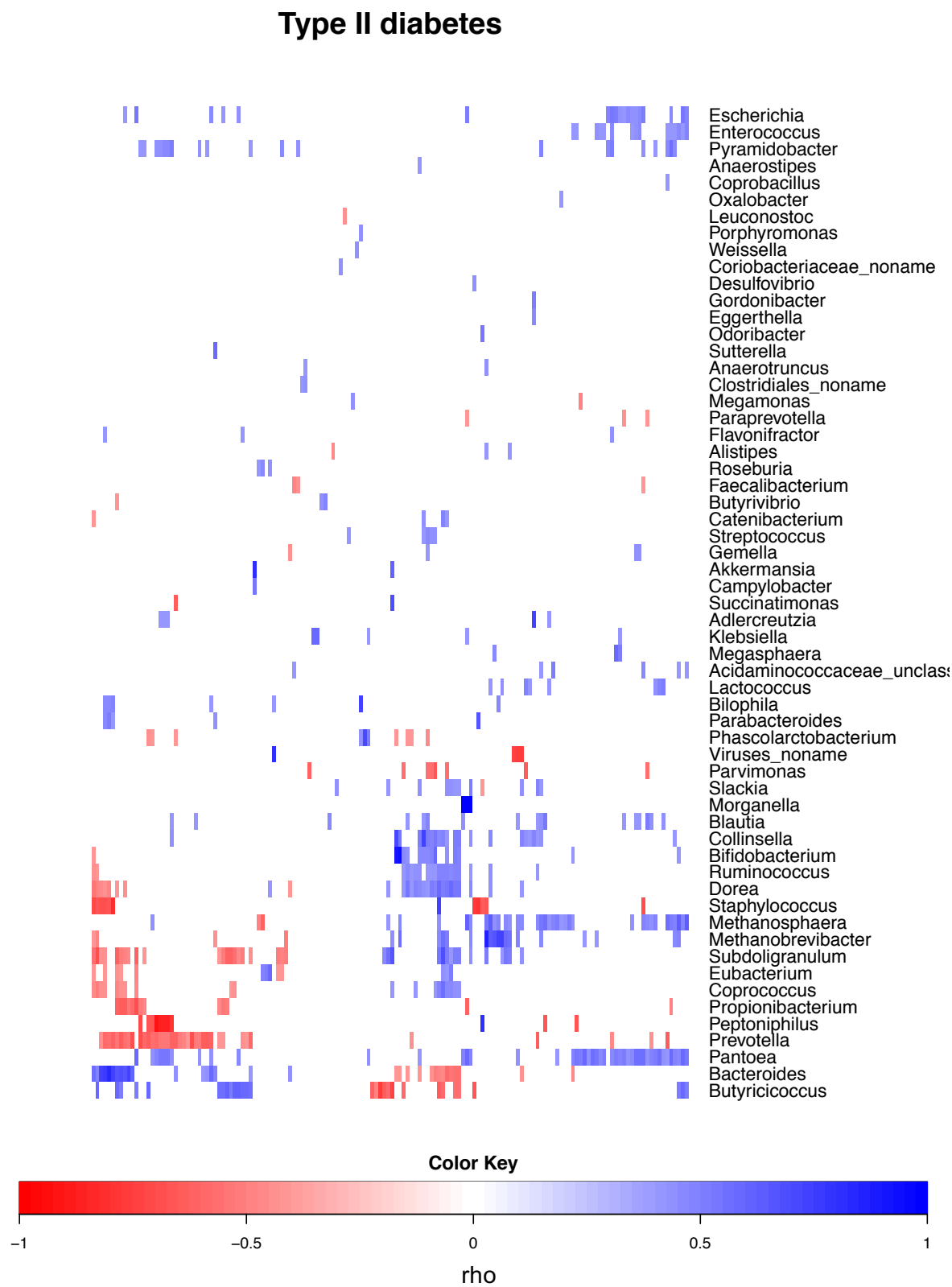

Figure S2 G

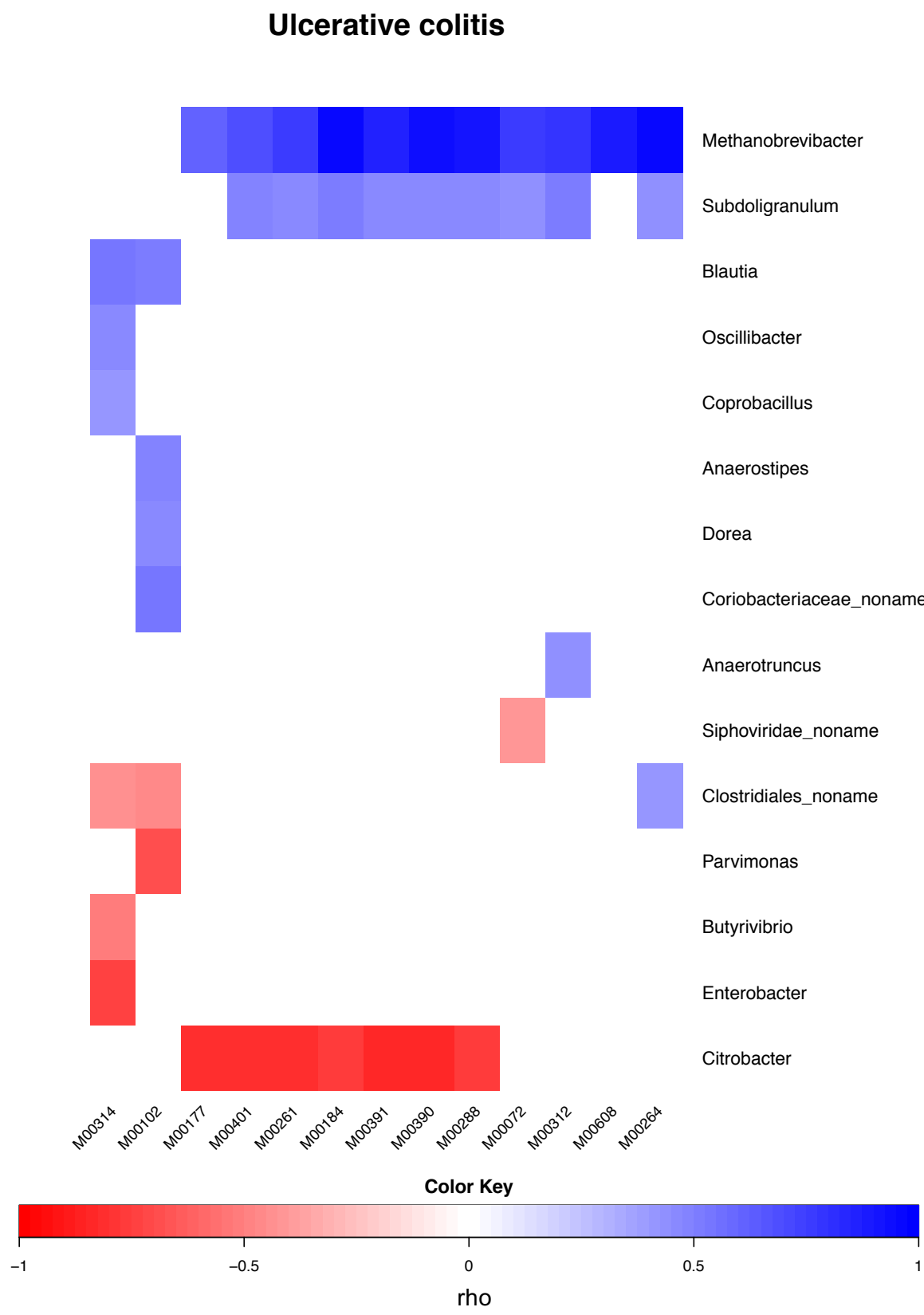

Supplement: FIG S2 [file mSystems.00332-18-sf002.pdf]
